# Shifting supply chain interdependencies among global automakers

**DOI:** 10.3389/fdata.2026.1886902

**Published:** 2026-07-23

**Authors:** Hiromitsu Goto, Wataru Souma

**Affiliations:** 1 Faculty of Information Engineering, Kanazawa Gakuin University, Kanazawa, Japan; 2 Faculty of Data Science, Rissho University, Kumagaya, Japan

**Keywords:** automotive industry, community detection, computational social science, electrification, graph mining, interfirm network, supply chain

## Abstract

Against the backdrop of electrification and supply chain resilience, the global automotive industry is in an era of great transformation. Using component supply information provided by MarkLines, this study investigated the impact on inter-firm dependencies among major global automakers from 2018 to 2024. Specifically, it analyzed changes in the community structure of the interdependency network between manufacturers, based on common suppliers for each model year and component category. The results revealed the impact of electrification: a reduction of roughly two-thirds (about 64%) in transactions for internal combustion engine (ICE) powertrains and an approximately eight-fold increase in e-powertrain transactions. Furthermore, the results of community detection also revealed a structural reorganization: while geographical clustering among manufacturers intensified for ICE components, new cross-border interdependencies formed for e-powertrain components, leading to the fragmentation of the traditionally integrated Japan-US-Europe bloc and the emergence of a China-centric ecosystem. This study provides new empirical evidence on the structural realignment of the global automotive value chain, offering important implications for management strategy and industrial policy in an era of great technological and geopolitical change.

## 1 Introduction

The global automotive industry is in the midst of a once-in-a-century paradigm shift, situated at the intersection of two powerful forces: the technological disruption of connected, autonomous, shared and service, electric (CASE) and a profound geopolitical realignment. The transition to electric vehicles (EVs) is not only redefining product architecture but also fundamentally reconfiguring the intricate web of production and supply that underpins the entire industry (Jagani et al., 2024; International Energy Agency, 2024). This transformation significantly impacts the structure of the automotive global value chain (GVC), which reshapes the industrial dynamics and regional configurations (Sturgeon et al., 2008; Ramos and Ruiz-Gálvez, 2024). Furthermore, recent disruptions such as the COVID-19 pandemic and semiconductor shortages exposed the vulnerabilities of global supply chains, elevating “resilience” from a corporate operational concern to a paramount national strategic imperative (Eldem et al., 2022; Mohammad et al., 2022; Seong et al., 2025).

Traditional hierarchical [Original equipment manufacturer (OEM) → Tier-1 → Tier-2] or linear supply chain models are insufficient for comprehending this complex environment. In recent years, the network analysis of interfirm transaction data has been increasingly recognized as a powerful tool for clarifying dynamic relationships within industrial structures (Inoue and Todo, 2019; Inoue et al., 2023; Fujiwara and Aoyama, 2010). Research on the automotive industry revealed complex dependencies between suppliers and manufacturers (Veloso and Kumar, 2002; Brintrup et al., 2012; Kito et al., 2014). However, a considerable amount of this prior work has been limited to static structural analyses at specific points in time or in particular regions, or it has focused on the aggregate analysis of the industry. There remains a scarcity of research that tracks the dynamic evolution of structural changes for each component category over time. The horizontal, indirect network of interdependencies among OEMs mediated by their shared reliance on a common pool of suppliers, has gained increasing importance in the current context of economic security. This overlooked structure is crucial for revealing systemic risks (Buldyrev et al., 2010; Kiparisov and Folberth, 2026), technological alliances, and competitive blocs increasingly shaped by geopolitical fragmentation and policy intervention (Karbevska et al., 2026; Klimek and Fooladi, 2026).

Such discussions on the transformation of network structures have been prominent in Japan since the early 2000s. The concept of “modularization,” as advanced by scholars such as Aoki et al. (2002) and debated in forums such as RIETI's “Economic Policy Review,” challenged the traditional “integral” (or suriawase) architecture that had long been a source of Japanese manufacturing strength. This theory suggested the necessity of moving away from fixed *keiretsu* systems toward a more open industrial architecture that can flexibly procure superior external technologies (modules) through standardized interfaces, and provided the intellectual foundation for the coming structural change.

Matous and Todo (2015) empirically demonstrated that the traditional *keiretsu* system within the Japanese domestic auto industry was gradually disassembling between 2006 and 2011, revealing a dynamic where OEMs achieved productivity gains by diversifying their supplier base [i.e., “opening” their procurement (Fujimoto, 2001)]. However, this prior research primarily focused on “efficiency” dynamics within Japan's “domestic” and “direct transaction” networks. How this “opened” market was subsequently reorganized on a “global” scale under the new mega-trends of electrification and geopolitical risk, and how “indirect interdependency” relationships among firms evolved, remains insufficiently explored. This paper aims to fill this critical research gap.

Therefore, this paper primarily aims to empirically map and analyze the structural evolution of the global automotive OEM interdependency network during the critical period of accelerated transformation from 2018–2024. We construct an OEM–OEM network where edge weights are defined by the number of common suppliers, using transaction data from the MarkLines information platform. Then, we apply community detection algorithms to trace the formation and dissolution of industrial blocs. Rather than proposing a new algorithm or metric, our contribution is empirical: we assemble a category-resolved, multi-year OEM interdependency network from a large proprietary dataset and use established community-detection methods to isolate how electrification reorganizes inter-OEM structure. Through this analysis, this paper provides novel, data-driven evidence of a fundamental realignment in the global production system, which is characterized by the fragmentation of the traditional Japan–US–Europe bloc and emergence of a China-centric ecosystem. Furthermore, it highlights starkly divergent evolutionary paths of supply chains for legacy internal combustion engine (ICE) technologies vs. emerging e-powertrain technologies, isolating the specific effect of electrification on inter-firm relationships. These findings offer important implications to understand industrial evolution, corporate strategy, and public policy in an era of technological and geopolitical change.

The remainder of this paper is organized as follows. Section 2 describes the data source and provides a descriptive analysis of the changing supplier market. Section 3 outlines the analytical framework, which comprises the method for constructing the OEM interdependency network and identifying industrial blocs via community detection. Section 4 presents the results and discusses the structural evolution of both aggregate and component-specific networks. Finally, Section 5 concludes the paper, discussing its implications and future research directions.

## 2 Data and descriptive analysis

### 2.1 Data source: MarkLines information platform

The empirical foundation of this study is a comprehensive dataset of automotive supply chain relationships sourced from the MarkLines information platform (MarkLines Co., Ltd., 2025), a globally recognized database providing detailed industry information. MarkLines data systematically track the suppliers that provide the specific part for a given vehicle model and model year, making its granularity and global coverage an invaluable resource to map production networks. The component suppliers for a given vehicle model are fixed after the start of production and subject to few major changes, which makes model-year data a valid proxy for analyzing temporal shifts in the industrial structure. Further it has been utilized in other academic research to analyze the structure of the global automotive supply chain.

The dataset provider, MarkLines, collects this proprietary supply chain information through a multifaceted research methodology. This information is not aggregated or made public, and it is instead gathered via a multi-angle approach that includes direct sourcing from industry exhibitions, targeted hearings, and analysis of press releases. The firm maintains global research bases across Europe, the Americas, and Africa, which enables it to provide data on mass-production vehicles worldwide. A key feature is its “300 parts supply chain” content, that is updated daily (e.g., ~48,000 updates in 2024), and possesses a high degree of currency, which includes information on models up to the 2027 model year. However, it is necessary to acknowledge the scope of data and the limitations. The research focuses on Tier-1 suppliers in the global market. As data are gathered through this diligent, ground-up investigation, there is no guarantee that it is comprehensive for every component across all models. The volume of available information may vary by part category and market.

For this analysis, we use a snapshot of the database comprising 277,157 unique supply linkages as of August 2025, focusing on the relationships of the top 25 global OEM groups (ranked by their number of recorded supply relationships) and their Tier-1 suppliers from 2018–2024. Importantly, the data indicate only the presence of a sourcing relationship and do not reflect the volume, monetary value, or strategic criticality of individual transactions. Consequently, throughout this study a shared-supplier link is weighted by the *number* of common supply relationships, which treats a low-value component (e.g., a sensor) identically to a high-value one (e.g., a battery pack); the resulting network therefore captures structural overlap in sourcing rather than economic dependence. This is a deliberate limitation of the present design, to which we return in Section 5. This study utilizes nine part classifications adopted in the MarkLines' component supply information as-is, conducting analysis across all categories.

Several preprocessing steps were performed on the raw data to ensure the consistency and accuracy required for rigorous network analysis. These included entity resolution to standardize firm names, OEM grouping to aggregate individual vehicle brands into their parent corporate groups as shown in Supplementary Table S1, temporal slicing to create discrete annual datasets, and categorical filtering based on nine part categories: body, chassis, driveline, electrical and electronic, exterior, general small parts, ICE-powertrain, interior, and e-powertrain.

### 2.2 Descriptive analysis: shifting supplier landscape (2018–2024)

**Figure 1:**
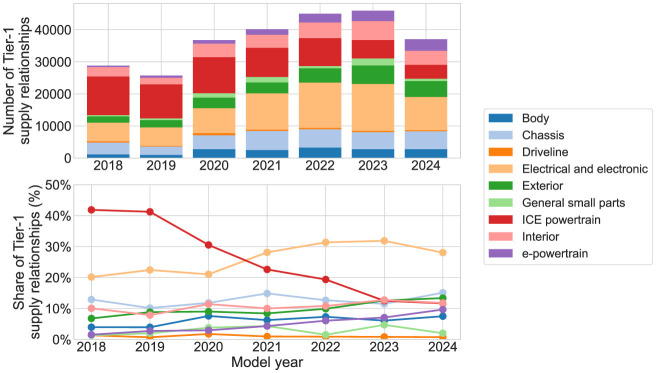
Supply relationship trends by component category, 2018–2024. The number of recorded supply relationships for ICE-powertrain components declined sharply, while e-powertrain relationships surged.

A preliminary descriptive analysis of the data reveals a profound and rapid transformation in the automotive supplier market between 2018 and 2024, which provides the quantitative context for the subsequent network analysis. The rise of electrification shows a dramatic technological substitution at the component level (see Figure 1). The number of recorded supply relationships for ICE-powertrain components plummeted by roughly two-thirds (about 64%) from 2018–2024, with its share of total transactions falling from ~42 to ~12%. In stark contrast, relationships for e-powertrain components surged roughly eight-fold, with their share rising from ~1.6 to ~9.7%, demonstrating a clear and rapid reallocation of sourcing activity toward electrification.

**Figure 2:**
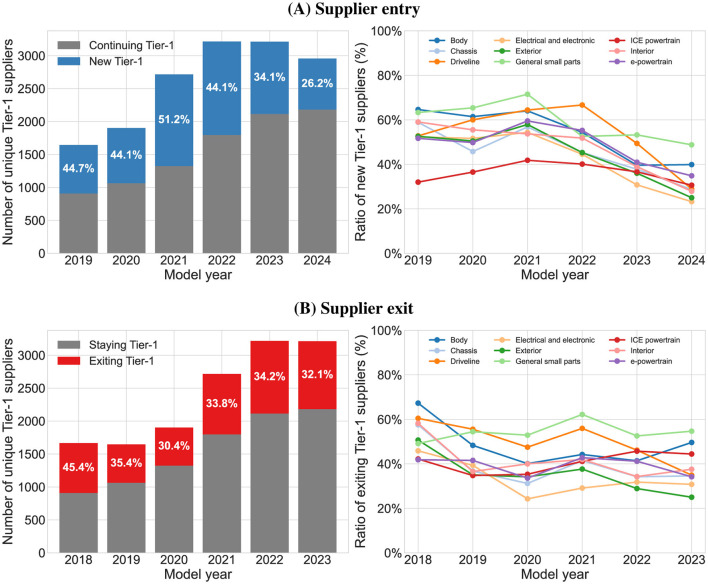
Year-over-year supplier churn analysis, 2018–2024. **(A)** New entrant counts **(left)** and ratios **(right)** by component category. **(B)** Exit counts **(left)** and ratios **(right)** by component category.

The period under review is characterized by exceptionally high market fluidity, as shown by supplier churn (see Figure 2A for entry and Figure 2B for exit). An analysis of year-over-year changes indicates a significant turnover in the supplier base. For example, in 2021, over half of the active suppliers were new entrants relative to the previous year, with this influx being most pronounced in the e-powertrain category. Concurrently, there is a high annual exit rate, with more than 30% of suppliers exiting the dataset each year, which is a trend particularly strong for suppliers in the ICE-powertrain category. This high rate of turnover suggests a destabilized competitive environment where the advantages of incumbent suppliers are being eroded, creating opportunities for new entrants, particularly those with expertise in electronics, software, and battery technology.

**Figure 3:**
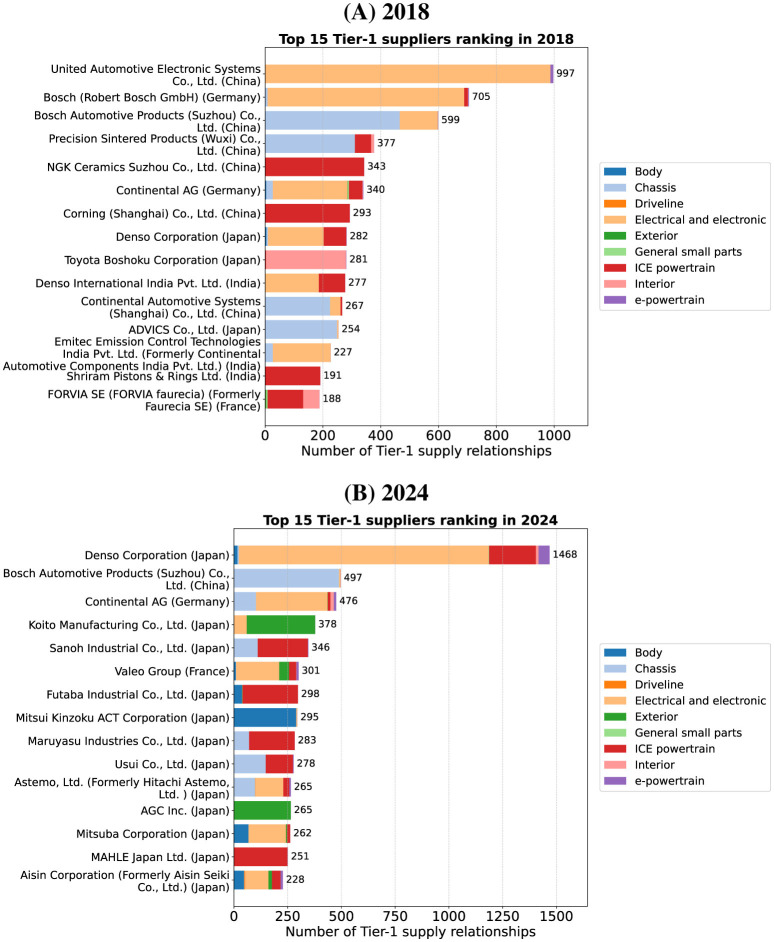
Top 15 hub suppliers by number of supply relationships in **(A)** 2018 and **(B)** 2024, broken down by component category.

This market churn is reflected in a significant reordering of the major “hub” suppliers of the industry [compare Figure 3A (2018) and Figure 3B (2024)]. A “hub” supplier refers to a firm with a high number of supply relationships in this dataset, and not one with a large transaction value. In 2018, the list of top suppliers was dominated by established European, Japanese, and American firms with deep roots in ICE technology, alongside their Chinese joint ventures. By 2024, the landscape shifted dramatically. Japan's Denso rose to the top, showcasing a stronger presence of Japanese firms that successfully pivoted to new technologies (e.g., Koito, Aisin) and notable rise of suppliers specializing in components for the entire vehicle architecture, not only the powertrain. The data highlight the strategic adaptation of incumbents and the growing influence of Chinese suppliers in the global market.

### 2.3 Descriptive analysis: OEM procurement strategies and positioning

**Figure 4:**
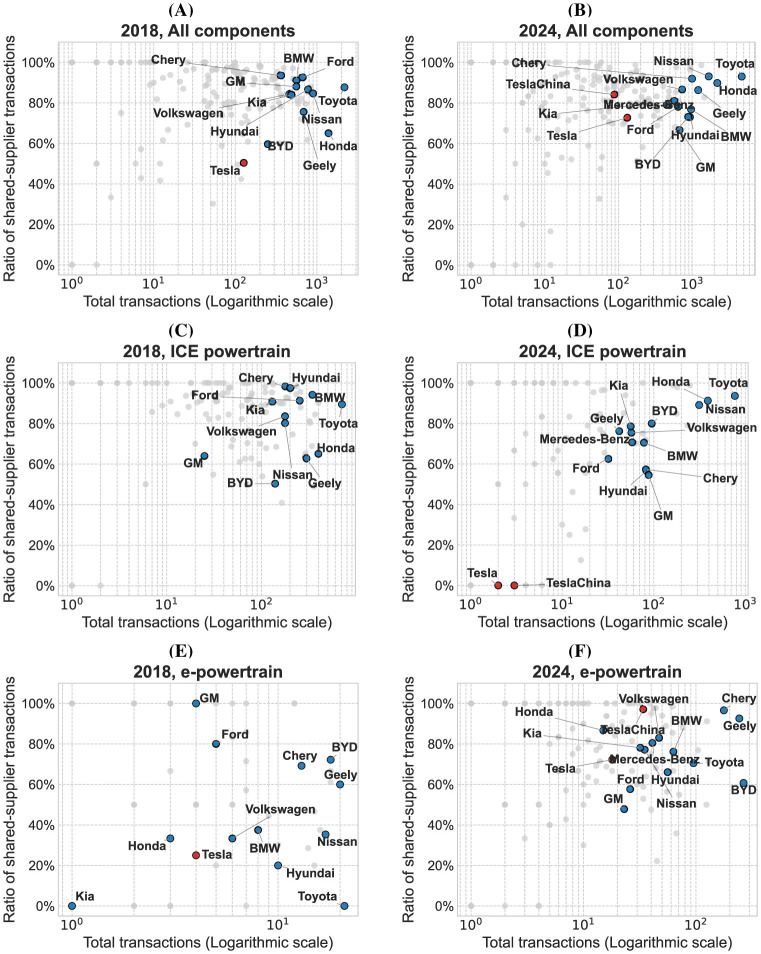
OEM procurement strategies: total transactions vs. shared supplier ratio. Each row compares 2018 **(left)** and 2024 **(right)** for **(A, B)** all components, **(C, D)** ICE-powertrain, and **(E, F)** e-powertrain.

Although the preceding section focused on trends in the supplier market, it is also crucial to survey the procurement strategies of the OEMs to understand the subsequent network analysis. This methodology proposed in this study captures interdependencies through shared suppliers, but not all OEMs adopt strategies predicated on such sharing. Figure 4 shows the total number of transactions and ratio of transactions with shared suppliers for all components, ICE-powertrain, and e-powertrain categories in 2018 and 2024. Results for remaining categories are provided in Supplementary Figures S1–S7. In these figures, the horizontal axis plots the total number of supplier transactions (logarithmic scale) of each OEM, whereas the vertical axis shows the ratio of transactions with shared suppliers. OEMs in the upper-right quadrant pursue a “horizontal collaboration” strategy, which engages with many suppliers, the majority of whom are shared with other OEMs. Conversely, OEMs in the lower-right quadrant, characterized by a large transaction volume but a low sharing ratio, may be pursuing a “vertical integration” strategy that emphasizes in-house production or exclusive supplier relationships.

Considering all component categories for 2024 (Figure 4B), traditional OEMs among the top 10 by transaction volume (blue dots), such as Toyota, Nissan, and Honda are positioned in the upper-right. In contrast, Tesla (red dot), which serves as a benchmark, already exhibited a relatively low sharing ratio in 2018 (Figure 4A), indicating an independent strategy. By 2024, BYD emerges in a highly distinctive position in the lower-right. Despite a very large number of supplier transactions, its sharing ratio is below 50%, clearly illustrating its strategy of strong vertical integration. This trend is pronounced in the e-powertrain category. In the 2024 e-powertrain scatter plot (Figure 4F), the sharing ratio of the BYD drops to ~ 35%, highlighting its unique strategy. Meanwhile, fellow Chinese OEMs Geely and Chery have a high number of transactions and a relatively high sharing ratio, which suggests that they are adopting a more horizontal, collaborative approach similar to that of traditional OEMs. Both Tesla and BYD thus occupy structurally atypical positions among major OEMs: their pronounced vertical integration means that, unlike conventional automakers whose interdependencies arise from broad shared-supplier overlap, their links to other firms are governed by a small number of strategically critical relationships. This distinction is important for interpreting their community membership in the network analysis that follows.

This descriptive analysis reveals a tectonic shift at the supplier level and diverse strategic positioning on the part of OEMs. The move to electrification is not merely changing the type of parts sourced but is redefining the very structure of the supplier market (who supplies and who exits). These fundamental changes in the supplier market and the differing procurement strategies of firms have reshaped interdependencies among manufacturers, which are formed through these shared suppliers, namely, the OEM–OEM network that is the subject of our analysis.

## 3 Analytical framework

### 3.1 Constructing the OEM interdependency network

We investigate the relational structure among global automakers. The analytical process begins by representing raw data as a bipartite network. This network, denoted as *G* = (*U, V, E*) includes two disjoint sets of nodes and edges that connect them. *U* = {*u*_1_, *u*_2_, ⋯ , *u*_*m*_} represents the set of *m* OEMs. *V* = {*v*_1_, *v*_2_, ⋯ , *v*_*n*_} represents the set of *n* Tier-1 suppliers. An edge *e*_*ij*_∈*E* exists between an OEM *u*_*i*_∈*U* and a supplier *v*_*j*_∈*V* if a supply relationship between them is recorded in the MarkLines database for a given year and part category. A one-mode projection is performed on the set of OEM nodes *U* as the primary interest of this study lies in indirect relationships among the OEMs. This projection transforms the bipartite network *G* into a unipartite, weighted network *G*′ = (*U, E*′, *W*). In this network, an edge exists between two OEMs if and only if they share at least one common supplier. This edge weight, *w*(*u*_*i*_, *u*_*k*_), serves as a measure of the intensity of their supply chain interdependency. The use of shared-supplier overlap as a proxy for interdependence follows an established literature on inter-firm and supply networks, in which a common supplier base is a recognized channel of correlated exposure and systemic risk (Brintrup et al., 2012; Kito et al., 2014; Buldyrev et al., 2010). We acknowledge, however, that shared suppliers may also arise from the prevalence of dominant global vendors, industry standardization, or common regulatory requirements; the measure therefore captures *potential* structural interdependence rather than confirmed strategic dependence.

### 3.2 Identifying industrial blocs via community detection

With the weighted OEM interdependency network *G*′ constructed in this study, the next step is to uncover its underlying mesoscale structure; the organization of nodes into groups or clusters are more densely connected internally than they are to the rest of the network. This process is known as community detection, which aims to partition the nodes of the network (OEMs) into a set of non-overlapping communities, revealing the de facto industrial blocs or ecosystems that exist within the global automotive industry. Therefore, this study employs a widely used class of methods based on modularity maximization. Modularity is a quality metric that measures the strength of a network's division into communities. As our primary method we use the greedy modularity-maximization algorithm of Clauset et al. (2004) (the Clauset–Newman–Moore, or CNM, method), an agglomerative optimizer that is deterministic and is therefore free of the run-to-run and seed dependence that can affect stochastic optimizers. To verify that the reported structures are not artifacts of a particular algorithm, we additionally compare the resulting partitions with those obtained from the Louvain (Blondel et al., 2008) and Leiden (Traag et al., 2019) algorithms, and we report partition stability and statistical significance in Section 4.4. The output of this process is a partition of the set of OEM nodes *U* into a set of communities *C* = {*C*_1_, *C*_2_, ⋯ , *C*_*k*_}. Each OEM is assigned to exactly one community. This analysis is performed independently for each of the constructed networks for each model year from 2018–2024 (e.g., All Parts 2018, All Parts 2019, ⋯ , All Parts 2024, and similarly for each part category). To characterize the resulting communities objectively rather than by inspection, we report for each community the share of member firms by headquarters country at both the corporate-group level and the operating-entity (Maker) level (Tables 1–4); the regional labels used in the discussion below (e.g., “Japanese,” “European,” “China-centric”) are assigned on the basis of these composition measures.

**Table 1:** Community features for all components in 2018.

Index (size)	Top 5 makers (by total shared suppliers)	Group HQ country ratio	Maker HQ country ratio
1 (30)	Nissan (579), Ford (511), Toyota (476), Honda (449), Audi (352)	Japan (0.31), Germany (0.27)	Japan (0.27)
2 (21)	SAIC GM (476), Geely (423), Great Wall (344), Changan Ford (335), BMW Brilliance (319)	China (0.50), USA (0.25)	China (1.00)
3 (14)	Dongfeng Nissan (546), GAC Honda (358), FAW Toyota (296), GAC Toyota (276), Dongfeng Renault (226)	Japan (0.85)	China (1.00)

Communities with size ≥2 are listed. Only HQ country ratios ≥20% are shown.

## 4 Results: evolving structure of global automotive supply chains

This section examines the structural evolution of the aggregate interdependency network, encompassing all component categories from 2018–2024, and presents a comparative analysis of networks for legacy (ICE-powertrain) and emerging (e-powertrain) technologies for isolating the specific impact of electrification.

### 4.1 Aggregate network: fragmentation and realignment (2018–2024)

The community detection analysis performed on the aggregate network reveals a dramatic structural transformation of the global automotive industry between 2018 and 2024, which is characterized by the fragmentation of the established industrial order and simultaneous consolidation of a new, powerful ecosystem.

In 2018, the OEM interdependency network exhibited a broadly bipolar structure, as detailed in Table 1. The largest group, Community 1, was a highly integrated bloc of 30 OEMs that included the majority of established Japanese, American, and European automakers such as Nissan, Ford, and Toyota, which represent the traditional core of the global industry. The second major group, Community 2, was distinctly Chinese-centric and composed of 21 firms led by joint ventures such as SAIC GM and domestic champions such as Geely. A third, smaller community (Community 3), centered on Chinese joint ventures of Japanese firms, acted as a bridge between the two larger blocs. This structure reflected an industry where a globalized incumbent core coexisted with a large but relatively distinct Chinese production system.

By 2024, this structure had undergone a radical realignment. The most significant finding, shown in Table 2, is the fragmentation of the formerly monolithic incumbent bloc, and the concurrent consolidation and expansion of the Chinese-centric ecosystem. The 2024 analysis identified three major communities with a fundamentally different composition. The Chinese-centric community (Community 1) grew significantly in size and internal cohesion, absorbing numerous players, with its composition now overwhelmingly Chinese, which is consistent with a move toward a more regionally concentrated supply base. Meanwhile, the traditional incumbent bloc fractured into two distinct communities: one centered on European, North American, and Korean automakers (Community 2), led by firms such as BMW, Mercedes-Benz, GM, and Ford, and another composed of the major Japanese OEMs (Community 3), such as Nissan, Toyota, Honda, and Mazda. Notably, the major U.S. automakers now group with the European-led community rather than with their Japanese counterparts, leaving the Japanese OEMs as a distinct bloc.

**Table 2:** Community features for all components in 2024.

Index (size)	Top 5 makers (by total shared suppliers)	Group HQ country ratio	Maker HQ country ratio
1 (34)	Geely (524), Chery (452), Great Wall (390), BYD (354), SAIC GM (245)	China (0.34), Japan (0.22)	China (0.97)
2 (33)	BMW (1124), GM (903), Ford (877), Audi (866), Mercedes-Benz (849)	Netherlands (0.36), Germany (0.27)	—
3 (11)	Nissan (1567), Toyota (1447), Honda (1405), Mazda (1070), Mitsubishi (1047)	Japan (0.88)	Japan (0.73)

Communities with size ≥2 are listed. Only HQ country ratios ≥20% are shown.

This evolution from a bipolar to a tripolar structure is consistent with a broad realignment of global automotive supply chains. We emphasize that our network measures overlap in supplier bases; it does not by itself establish strategic intent, economic decoupling, or industrial-policy causation, and the interpretations that follow should be read in that descriptive light. The consolidation of the Chinese community into a larger and more cohesive bloc is consistent with the growth of China's domestic supply capacity in the EV domain. The fracturing of the incumbent bloc is consistent with Western and Japanese firms reconfiguring their supply networks, potentially driven by a combination of factors: a response to Chinese competition, an effort to build more resilient and regionalized supply chains in the face of geopolitical uncertainty, and divergent technological pathways being pursued by different regional actors. In structural terms, the network has reorganized along clearer regional and corporate lines.

### 4.2 Divergent paths: legacy consolidation vs. new ecosystem formation

**Figure 5:**
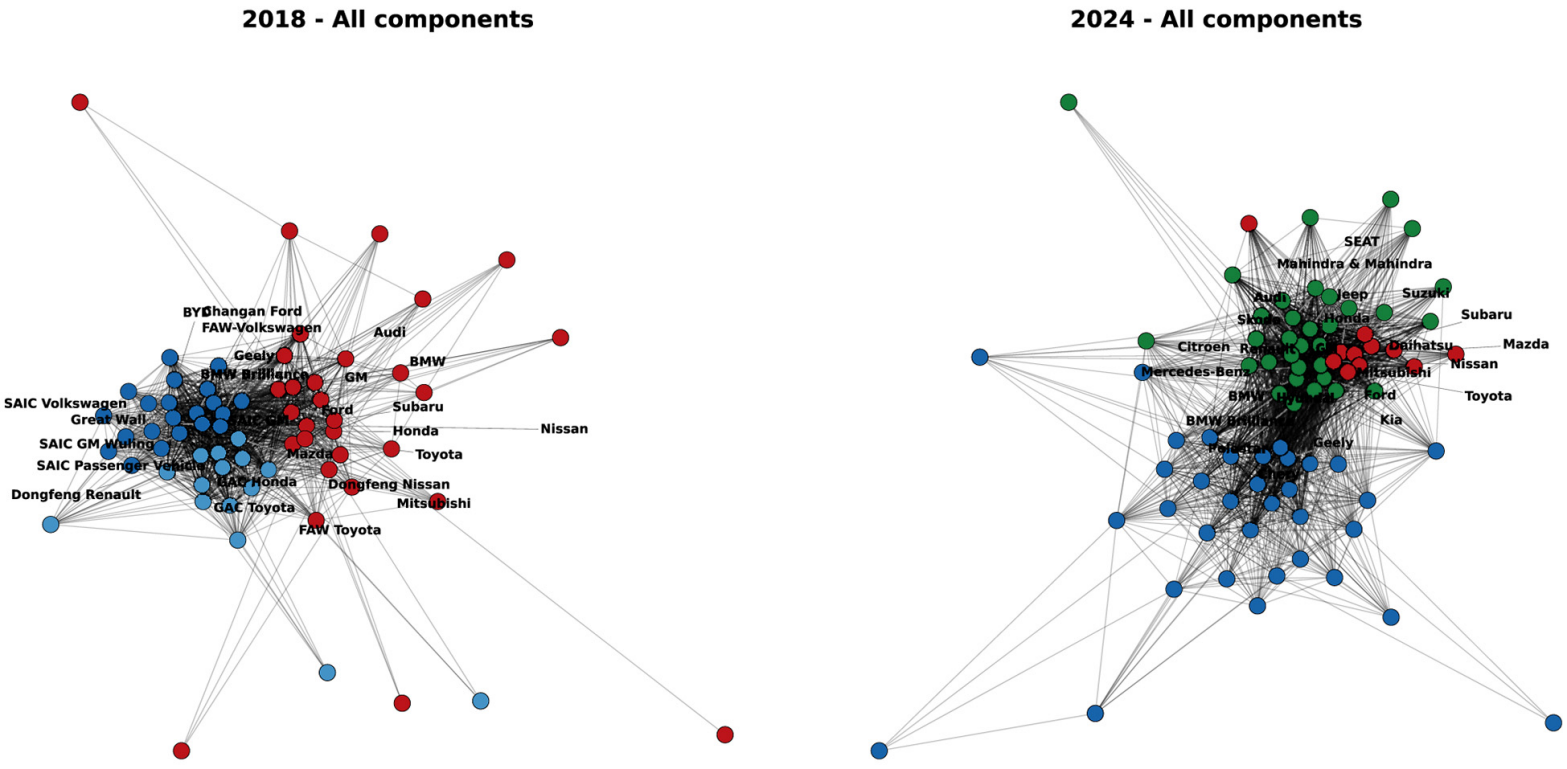
OEM interdependency network for all components, 2018 vs. 2024. Communities are detected by the greedy modularity-maximization (CNM) algorithm. Each node's hue indicates the dominant headquarters region of its community (red: Japan; blue: China; green: Europe; purple: Korea; orange: North America); where a region contains more than one community, shade distinguishes them (darker = larger, ordered as in the community tables). The same color scheme is used in the temporal overview.

**Figure 6:**
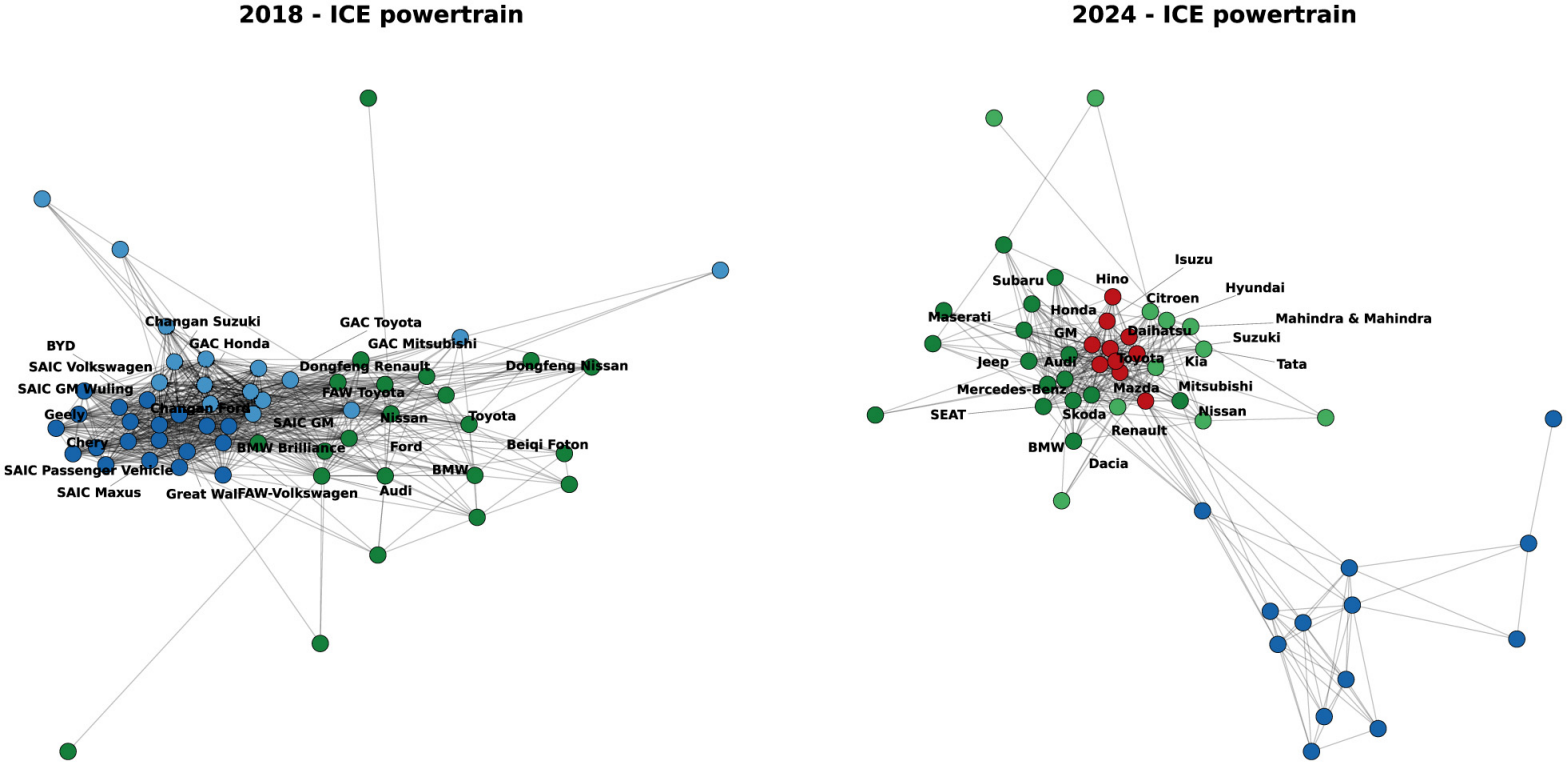
OEM interdependency network for ICE-powertrain components, 2018 vs. 2024. Nodes are colored by community as in Figure 5 (hue = community's dominant headquarters region; shade distinguishes multiple communities of the same region, darker = larger).

**Figure 7:**
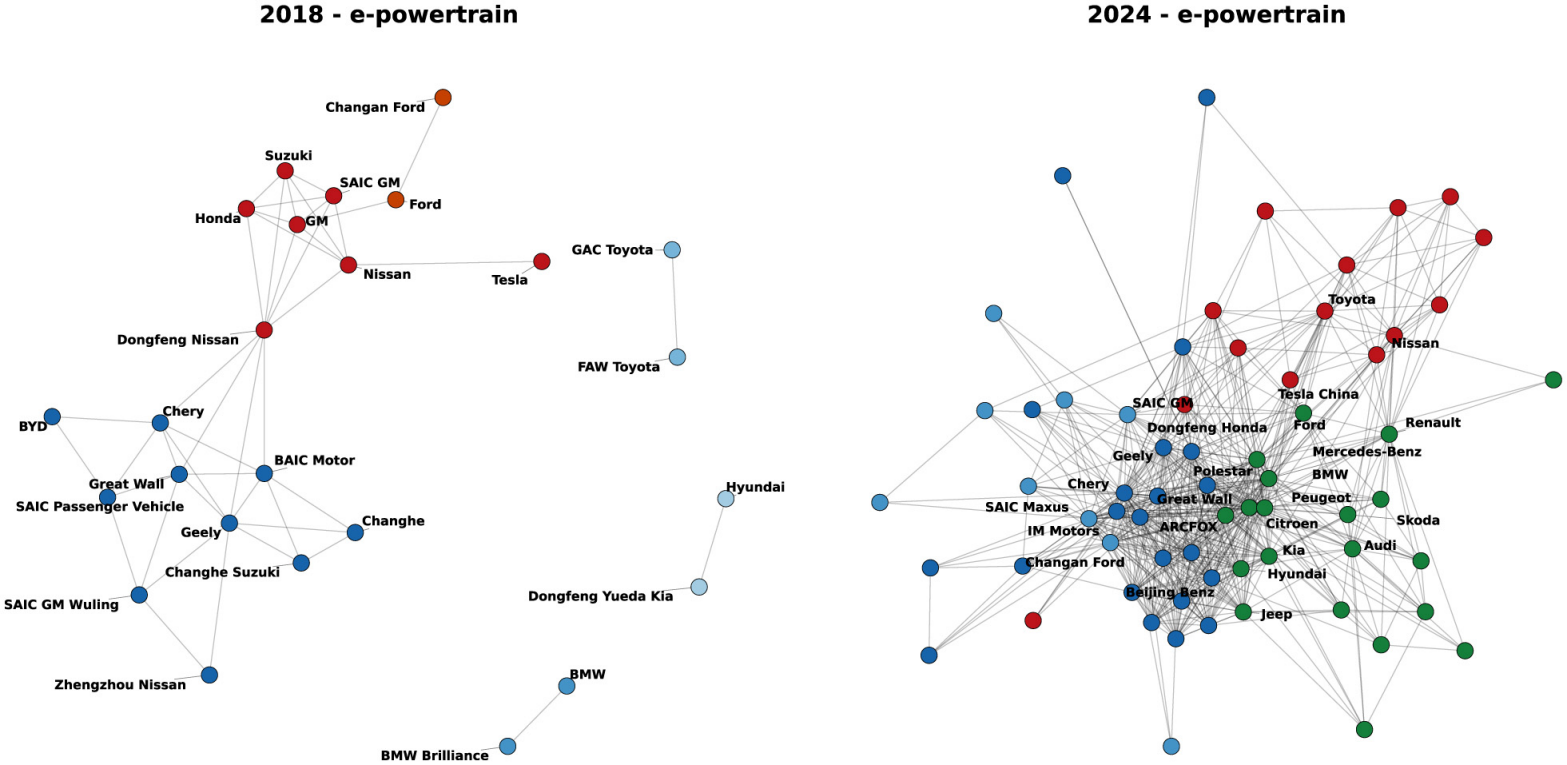
OEM interdependency network for e-powertrain components, 2018 vs. 2024. The network evolved from a sparse, fragmented state in 2018 to a dense, globally interconnected system by 2024. Nodes are colored by community as in Figure 5 (hue = community's dominant headquarters region; shade distinguishes multiple communities of the same region, darker = larger).

To understand the primary drivers of this aggregate-level restructuring, the analysis was extended to individual component technologies. This paper presents network visualizations for 2018 and 2024 for all components (Figure 5), ICE-powertrain (Figure 6), and e-powertrain (Figure 7), with the corresponding tables of community characteristics. Network visualizations and community characteristics for remaining categories are provided in Supplementary Figures S8–S14 and Supplementary Tables S2–S19. This granular category-specific analysis reveals diverse patterns of interdependence obscured in the aggregate network view. Mature mechanical components such as body, chassis, and exterior maintain relatively stable regional blocs across 2018–2024, with recognizable Japanese, European, and Chinese communities persisting throughout. In contrast, the electrical and electronic network exhibits the most pronounced densification and re-ordering of any non-powertrain category—driven by the proliferation of electronic control units, sensors, and battery-management systems associated with electrification and the software-defined vehicle—forming new technological-partnership alliances that cut across traditional regional lines. These category-level results, detailed in the Supplementary material, demonstrate that the nature of co-dependency varies systematically by component type. However, the most striking divergence is observed between the legacy ICE-powertrain sector and emerging e-powertrain sector.

The comparative analysis for 2024 detailed in Tables 3, 4 reveals starkly divergent evolutionary paths for the legacy ICE-powertrain sector and emerging e-powertrain sector. The shift in product architecture from mechanical to electrical systems is mirrored by a fundamental shift in the architecture of the corresponding supply networks.

**Table 3:** Community features for ICE-powertrain in 2024.

Index (size)	Top 5 makers (by total shared suppliers)	Group HQ country ratio	Maker HQ country ratio
1 (16)	Audi (90), BMW (81), Skoda (81), Mercedes-Benz (75), GM (70)	Netherlands (0.44), Germany (0.31)	USA (0.25)
2 (13)	SAIC passenger vehicle (16), BYD (16), Chery (15), Great Wall (13), BMW Brilliance (12)	China (0.42), USA (0.33)	China (1.00)
3 (11)	Kia (51), Renault (45), Mahindra & Mahindra (39), Hyundai (36), Citroen (34)	Netherlands (0.36), S. Korea (0.27)	India (0.36)
4 (10)	Toyota (208), Nissan (199), Mitsubishi (163), Honda (155), Mazda (122)	Japan (1.00)	Japan (0.80), Thailand (0.20)

Communities with size ≥2 are listed. Only HQ country ratios ≥20% are shown.

**Table 4:** Community features for e-powertrain in 2024.

Index (size)	Top 5 makers (by total shared suppliers)	Group HQ country ratio	Maker HQ country ratio
1 (22)	Geely (88), Chery (66), Great Wall (58), Tesla China (51), Polestar (49)	China (0.38)	China (0.91)
2 (20)	BMW (90), Mercedes-Benz (69), Peugeot (61), Ford (55), Citroen (51)	Germany (0.30), Netherlands (0.30)	Germany (0.20)
3 (14)	Toyota (41), Nissan (36), Mazda (29), BYD (29), GAC Toyota (22)	Japan (0.82)	Japan (0.57), China (0.21)
4 (9)	IM motors (49), SAIC Maxus (45), SAIC GM (37), SAIC Volkswagen (23), SAIC passenger vehicle (18)	China (0.33), USA (0.22), Germany (0.22)	China (1.00)

Communities with size ≥2 are listed. Only HQ country ratios ≥20% are shown.

The network of interdependencies for ICE-powertrain components indicates retrenchment and regional consolidation. By 2024, the ICE network reorganized into much more distinct, geographically defined communities. As shown in Table 3, this includes a clear Japanese community (Community 4) comprising firms such as Toyota, Nissan, and Honda; a European-centric community (Community 1) led by German luxury brands; and a Chinese community (Community 2) of domestic firms and their joint ventures. This evolution suggests a consolidation of legacy supply chains. As ICE technology reaches maturity and new investment is overwhelmingly directed toward electrification, automakers appear to be reinforcing their most established, efficient, and geographically proximate supply networks for these components. There is less incentive to forge new, complex global partnerships in a technologically stable but declining market segment.

In sharp contrast, the analysis of the e-powertrain network presents a dramatic narrative of the birth of an entirely new industrial ecosystem. A sparse and fragmented network in 2018 coalesced into a dense, highly interconnected global system by 2024. It has novel structure, as detailed in Table 4. First, a massive, dominant Chinese-centric community (Community 1) emerged; this community was far larger and more diverse than its ICE counterpart. This includes not only Chinese domestic giants but also the Chinese operations of global players such as Tesla, indicating China's central role as both a market and a production hub for EVs. Second, a distinct European-Korean community (Community 2) formed, led by firms such as BMW, Mercedes-Benz, and Hyundai/Kia, suggesting a strategic alignment in developing a non-Chinese EV supply base. Most strikingly, a new Japanese-led community (Community 3) appeared, anchored by Toyota, Nissan, Honda, and critically including the Chinese battery and EV behemoth, BYD.

**Figure 8:**
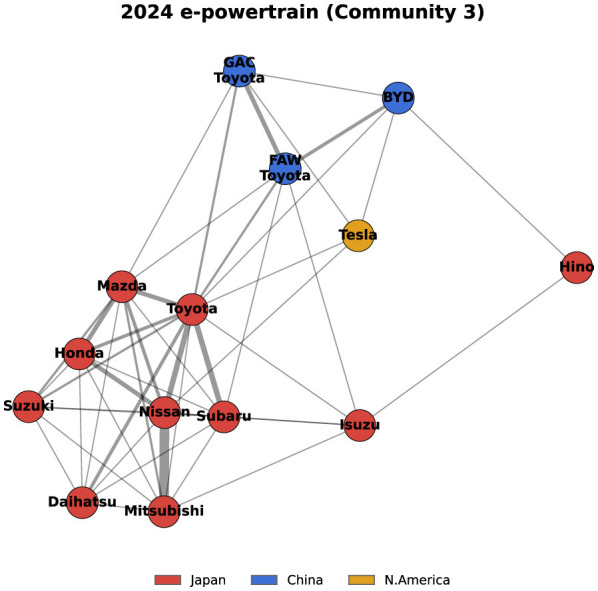
Detail of Community 3 in the 2024 e-powertrain network, showing the Japanese-led community that includes BYD. In contrast to Figures 5–7, where a node's hue denotes its community's dominant region, here each node is colored by its own headquarters region (red: Japan; blue: China; gold: North America). This highlights that BYD and the Chinese joint-venture operations (blue) are embedded among the Japanese OEMs (red) that constitute the community.

Figure 8 and Table 5 detail the structure of Community 3 in the 2024 e-powertrain network. BYD's inclusion in this Japanese-centric cluster is driven by its specific ties with Toyota's Chinese joint ventures, rather than global integration. As BYD is highly vertically integrated with few shared suppliers, these specific links become structurally significant. The table reveals a sharp contrast: while Toyota (Global) shares only a standardized thermal management supplier (Zhejiang Sanhua) with BYD, its joint venture, FAW Toyota, sources core powertrain components directly from BYD's subsidiary, FinDreams. This indicates that Toyota maintains its proprietary technologies for key EV components globally and is compelled to deepen interdependence with BYD in its Chinese production (FAW Toyota) to meet local market demands and ensure competitiveness.

**Table 5:** Makers sharing common suppliers with BYD and their products in Community 3 (2024 e-powertrain).

Maker	Common supplier	Products
FAW Toyota	FinDreams Powertrain Co., Ltd. Changsha branch	3-in-1 electric drive system (motor, inverter and reducer; 200 kW), Traction motor (3-in-1 electric drive system; 200 kW)
FAW Toyota	Wuwei FinDreams Battery Co., Ltd.	Lithium iron phosphate battery cell (blade), Lithium iron phosphate battery pack
GAC Toyota	Zhejiang Sanhua Automotive Components Co., Ltd.	Battery cooling plate
Toyota	Zhejiang Sanhua Automotive Components Co., Ltd.	Battery cooling plate
Tesla	Zhejiang Sanhua Automotive Components Co., Ltd.	Battery cooling system
Hino	Shinry Technologies Co., Ltd.	On-board charger

Community 3 is listed among the community characteristics shown in Table 4, and the network is shown in Figure 8.

This structure signifies the creation of a new global supply chain order for EVs. The presence of BYD, which is a direct competitor to Japanese OEMs in the final vehicle market, within the Japanese e-powertrain community is a notable illustration of this new reality. This indicates a critical cross-national dependency of Japanese automakers on Chinese technology and production capacity for essential EV components, particularly batteries. This pattern is consistent with widely documented developments in the EV battery industry, in which Chinese battery and e-drive suppliers have become primary sources for global automakers (International Energy Agency, 2024; Jagani et al., 2024); a full quantitative cross-validation against trade and battery-shipment statistics is, however, beyond the scope of the present network analysis and is left for future work. This finding suggests a potential tension between the strategic imperative for individual firms to secure critical technology and the long-term national industrial policy goal of supply chain independence. In the e-powertrain domain, national and regional boundaries are far more porous, and technological leadership creates interdependencies that transcend traditional competitive lines.

### 4.3 Temporal continuity of community structure

**Figure 9:**
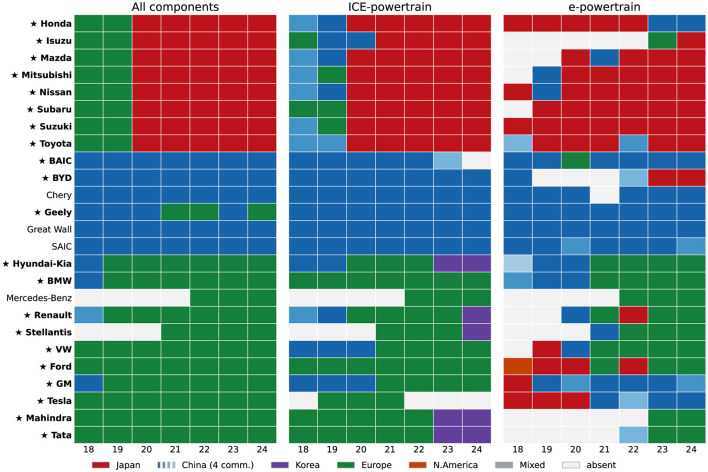
Community membership of the 25 OEM groups over 2018–2024 for all components (**left**), ICE-powertrain (**center**), and e-powertrain (**right**). Each cell's hue indicates the dominant headquarters region of the community to which the group's principal maker belongs in that year (red: Japan; blue: China; green: Europe; purple: Korea; orange: North America; gray: mixed; white: absent), using the same color scheme as Figures 5–7; where a region contains more than one community, shade distinguishes them (darker = larger). Cross-year community identity is tracked by membership overlap, so a hue remains stable while a group stays in the same evolving regional bloc and changes when it moves to a different bloc; a star (⋆) marks groups that change bloc in at least one network.

The comparisons presented above contrast two endpoints, 2018 and 2024. To verify that the structural realignment they reveal reflects a continuous evolution rather than an artifact of comparing two arbitrary snapshots, Figure 9 visualizes the community membership of every OEM group across all model years from 2018 to 2024. Each cell is colored by the dominant headquarters region of the community to which the group's principal maker belongs in that year; cross-year community identity is tracked by membership overlap (Jaccard) so that a change of color marks a genuine move between blocs. We present this as a descriptive visualization rather than a formal dynamic-network model.

For all components (Figure 9, left), the transition is visibly gradual. The integrated bloc that dominates 2018–2019 does not fracture abruptly; instead, the Japanese OEMs progressively consolidate into a distinct Japanese community (red) from around 2020 onward, while the China-centric bloc (blue) persists throughout and the European grouping (green) remains stable. Consistent with this visual continuity, the year-to-year stability of community membership is high—the adjusted Rand index between consecutive years rises from roughly 0.6 in 2018–2019 to about 0.95 by 2021–2022 (Section 4.4)—confirming that the tripolar structure emerges through an accretive process rather than a single discontinuous jump. The e-powertrain panel (Figure 9, right) is far more volatile, mirroring the rapid formation of the EV supply base: BYD is fragmentary or absent from the network in the sparse early years and only settles into the Japanese-led community in 2023–2024. The BYD finding is therefore a temporally emergent phenomenon—the crystallization of a new cross-border dependency—rather than a static feature of a single year. A full dynamic-network treatment [for example, dynamic stochastic block models or tensor decomposition, within the broader temporal-network program reviewed by Holme and Saramäki (2012)] lies beyond the empirical scope of this study and is left for future work (Section 5).

### 4.4 Robustness of the community structure

Because community detection can be sensitive to the choice of algorithm and to stochastic optimization, we assessed the robustness of the partitions reported above along three dimensions: modularity values, agreement across algorithms, and statistical significance against a null model. Table 6 summarizes the results for the four focal networks.

**Table 6:** Robustness of the community partitions for the four focal networks.

Network	Modularity *Q*	#Comm.	ARI vs. Louvain	ARI vs. Leiden
All components, 2018	0.31	3	1.00	1.00
All components, 2024	0.27	3	0.92	0.95
ICE-powertrain, 2024	0.33	4	0.94	0.94
e-powertrain, 2024	0.29	4	0.33	0.65

*Q* is the modularity of the primary (CNM) partition; #Comm. is the number of communities of size ≥2; ARI is the adjusted Rand index between the CNM partition and the Louvain (γ = 1.0, seed 42) and Leiden (seed 42) partitions.

First, all partitions attain modularity values in the range *Q*≈0.27–0.33, indicating clear community structure. Second, the partitions are highly consistent across modularity-maximization algorithms: the deterministic CNM partitions agree almost perfectly with both Louvain and Leiden for the aggregate networks (adjusted Rand index, ARI = 1.00 for 2018; 0.92–0.95 for 2024) and for the 2024 ICE-powertrain network (ARI = 0.94), and a 50-seed Louvain ensemble reproduces the CNM partition with mean ARI ≥0.92 in these cases. Third, the headline structures are statistically significant against a degree-preserving (curveball) null model: the 2018 bipolar and 2024 tripolar aggregate structures and the 2024 ICE-powertrain regional consolidation all yield *z*≈24–80 (*p*≈0.005). The accompanying temporal changes are likewise significant (the ICE decline and e-powertrain rise have Mann–Kendall *p*≈0.003–0.007 with bootstrap confidence intervals excluding the null).

The one case that is genuinely sensitive is the 2024 e-powertrain network, where the CNM partition agrees only moderately with Louvain (ARI = 0.33) and Leiden (ARI = 0.65), and the 2018 e-powertrain structure is not significant against the null (*z*≈1.2, *p*≈0.13), reflecting its sparsity. This sensitivity is concentrated in the membership of BYD (Section 4 and Table 5): across 100 Louvain seeds, BYD co-clusters with the Chinese operations of Japanese OEMs (FAW Toyota, GAC Toyota, Dongfeng Nissan) essentially always, but whether that BYD–joint-venture cluster *merges* with the parent Japanese OEMs varies. The deterministic modularity-maximization methods (CNM and the fast-greedy variant) and Leiden place BYD within the Japanese-led community, whereas Louvain at the default resolution tends to separate it. We therefore report BYD's position transparently: the underlying mechanism—BYD/FinDreams supplying batteries and e-drive components to Japanese OEMs' Chinese operations—is robust, while its exact community label is resolution-sensitive and rests on a small number of links under the count-based edge weighting (Section 2.1).

## 5 Conclusion

This study empirically mapped the evolving structure of interdependencies among global automotive OEMs during a period of unprecedented technological and geopolitical change. The analysis of the aggregate OEM network confirms a significant structural realignment: the traditionally integrated automotive bloc of Japanese, American, and European firms has fragmented, while a large, cohesive, and increasingly self-sufficient Chinese-centric ecosystem has risen and consolidated. The structure of industry has shifted from a bipolar model to a more multipolar configuration. The study reveals that the evolutionary paths for legacy and emerging technologies are starkly different. For ICE-powertrain components, the network has evolved toward greater regional consolidation. In sharp contrast, the e-powertrain network has rapidly evolved from a fragmented state to a dense, globally interconnected system. The structure of this new e-powertrain ecosystem is defined by novel, cross-national alliances driven by access to critical technologies, and it is exemplified by the inclusion of the Chinese giant BYD within the Japanese-led community, highlighting a critical dependency that transcends traditional competitive and national boundaries.

These findings carry significant implications for both corporate strategists and public policymakers. For managers, they underscore the urgent requirement for a technology-specific supply chain strategy. For ICE components, the focus should be on regional efficiency, while for EVs, the imperative is to secure access to new technologies and manage complex global dependencies. The network maps presented here can serve as a strategic risk assessment tool for identifying hidden vulnerabilities. For policymakers, this research provides empirical evidence relevant to concerns about geopolitical dependencies in new energy technology supply chains. The clear emergence of a dominant, Chinese-centric community in the global e-powertrain network highlights the formidable strategic challenge facing other nations and reinforces the rationale behind industrial policies aimed at building resilient, competitive, and diversified domestic supply chains for batteries, semiconductors, and other essential components.

This study has limitations that point toward promising avenues for future research. The network edge weights are based on the number of shared supply relationships not their economic value or strategic criticality. The analysis is confined to relationships between OEMs and their Tier-1 suppliers without capturing critical dependencies at lower tiers of the supply chain, such as for semiconductors and raw materials for batteries. Although this paper complements its comparative statics between 2018 and 2024 with a visualization of the year-over-year evolution of community membership (Section 4.3), it stops short of a formal dynamic-network model; a more detailed dynamic analysis—for example, one based on transition probabilities or dynamic stochastic block models—would help identify the specific trigger events (e.g., major alliances, policy shifts) behind structural change. These limitations inform a clear agenda for future work. This includes developing more sophisticated weighted network analyses that incorporate data on transaction value or technological importance. Furthermore, as the transformation of the industry continues, future analysis must consider the rise of the software-defined vehicle. A critical future challenge will be to expand the network model to include the role of new entrants from the technology sector, such as software and platform providers like Google, and to analyze their effect on the established ecosystem.

## Data Availability

The data analyzed in this study is subject to the following licenses/restrictions: the dataset is a proprietary commercial database provided by MarkLines Co., Ltd. (https://www.marklines.com) and is accessible only through a paid subscription. The authors used the data under a commercial license that prohibits redistribution to third parties. Therefore, the raw data cannot be made publicly available or shared by the authors. Researchers wishing to access the data must obtain a subscription directly from MarkLines. Requests to access these datasets should be directed to MarkLines Co., Ltd., https://www.marklines.com.
