# Supplementary material for: Shifting supply chain interdependencies among global automakers
Source: Front Big Data. 2026 Jul 23;9:1886902. doi: 10.3389/fdata.2026.1886902 (PMC13441850; doi:10.3389/fdata.2026.1886902)
Supplement: Supplementary file 1 [file Data_Sheet_1.pdf]

Supplementary Material: Shifting Supply Chain Interdependencies Among Global Automakers

1 OVERVIEW

This Supplementary Material provides detailed analyses that complement the main manuscript "Shifting Supply Chain Interdependencies Among Global Automakers." It contains (i) the complete mapping of original equipment manufacturer (OEM) brands to their corporate groups used throughout the study, (ii) category-specific procurement strategy scatter plots for component categories not presented in the main text, (iii) OEM interdependency network visualizations for each of the nine component categories, and (iv) community detection results for every combination of component category and model year (2018 and 2024). Together, these materials demonstrate that the structural patterns emphasized in the main text—horizontal collaboration versus vertical integration, geographical consolidation of legacy (ICE-powertrain) networks, and the emergence of a Chinese-centric electric-powertrain ecosystem—are consistent across, and extend well beyond, the subset of figures included in the main manuscript.

All figures in this Supplementary Material were generated using the same analytical pipeline and graphical conventions described in Section 2 and Section 3 of the main text. Networks were constructed from the MarkLines information platform snapshot (August 2025), filtered to the top 25 global OEM groups, projected onto a unipartite Maker–Maker network weighted by the number of shared Tier-1 suppliers, and partitioned via the deterministic greedy modularity-maximization (Clauset–Newman–Moore, CNM) community detection algorithm used as the primary method in the main text.

2 OEM GROUPING

Table S1 provides the full mapping of individual vehicle brands ("Makers") to their parent corporate groups ("Groups") used in the main analysis. The grouping aggregates joint ventures, regional subsidiaries, and historically related brands into unified economic decision-making units. For example, Toyota Group includes not only Toyota and its wholly-owned brands (Hino, Daihatsu) but also its Chinese joint ventures (FAW Toyota, GAC Toyota) and its Taiwanese production partner (Kuozui). Group-level aggregation is essential for analyzing strategic supplier-sharing behavior, because procurement decisions are typically coordinated at the group level rather than at individual brand levels. Similarly, Stellantis groups 15 brands spanning European, American, and Chinese operations, while BYD Auto consists of BYD and BYD Trucks. Post-2021 Stellantis formation and post-2022 Mercedes-Benz Group reorganization are reflected in the grouping.

Table S1: OEM Grouping used in this study.

Group (Group HQ Country)	Makers (Maker HQ Country)
Toyota Group (Japan)	Toyota (Japan), Hino (Japan), Daihatsu (Japan), FAW Toyota (China), GAC Toyota (China), Kuozui (Taiwan)

Continued on next page

Table S1 – *Continued from previous page*

Group (Group HQ Country)	Makers (Maker HQ Country)
VW Group (Germany)	Volkswagen Anhui (China), SAIC Volkswagen (China), Audi (Germany), Bentley (UK), SEAT (Spain), Lamborghini (Italy), FAW-Volkswagen (China), Skoda (Czech Republic)
Hyundai Kia Automotive Group (South Korea)	Kia (South Korea), Beijing Hyundai (China), Dongfeng Yueda Kia (China), Yueda Kia (China), Hyundai (South Korea)
GM Group (USA)	GM (USA), Wuling (China), SAIC GM Wuling (China), SAIC GM (China), Shanghai GM (China)
Stellantis (Netherlands)	DS Automobiles (France), Dongfeng Peugeot Citroen (China), Alfa Romeo (Italy), Dodge (USA), Chrysler (USA), Opel (Germany), Maserati (Italy), Ram Trucks (USA), Lancia (Italy), Citroen (France), GAC Fiat Chrysler (China), Peugeot (France), Jeep (USA), Fiat (Italy)
BYD Auto (China)	BYD (China), BYD Trucks (China)
Ford Group (USA)	Ford (USA), Jiangling Motors (China), Changan Ford (China)
Geely Holding Group (China)	LEVC (UK), Polestar (Sweden), Geely (China)
Honda (Japan)	Dongfeng Honda (China), GAC Honda (China), Honda (Japan)
Suzuki (Japan)	Changan Suzuki (China), Magyar Suzuki (Hungary), Changhe Suzuki (China), Suzuki (Japan)
Nissan (Japan)	Dongfeng Nissan (China), Zhengzhou Nissan (China), Nissan (Japan)
Chery Automobile (China)	JETOUR (China), Chery (China)
BMW Group (Germany)	BMW Brilliance (China), BMW (Germany), Rolls-Royce (UK)
Changan Automobile Group (China)	Deepal (China), Changan (China)
Renault (France)	Dongfeng Renault (China), Renault Korea (South Korea), Renault (France), Dacia (Romania)
Mercedes-Benz Group (Germany)	Daimler (Germany), smart (Germany), Fujian Benz (China), Beijing Benz (China), Daimler Truck (Germany), Mercedes-Benz (Germany)

Continued on next page

Table S1 – *Continued from previous page*

Group (Group HQ Country)	Makers (Maker HQ Country)
Tesla (USA)	Tesla China (China), Tesla (USA)
SAIC (China)	SAIC Passenger Vehicle (China), IM Motors (China), SAIC Maxus (China)
Mazda (Japan)	Changan Mazda (China), FAW Mazda (China), Mazda (Japan)
Tata Group (India)	Tata (India)
BAIC Group (China)	BAIC ORV (China), BAIC Motor (China), ARCFOX (China), Beiqi Foton (China)
Mahindra & Mahindra (India)	Mahindra & Mahindra (India)
FAW (China)	FAW (China), FAW Car (China)

3 CATEGORY-SPECIFIC PROCUREMENT STRATEGIES

Figures S1–S7 present OEM procurement scatter plots (total transactions vs. ratio of shared-supplier transactions) for the seven component categories that are not included in the main manuscript: body, chassis, driveline, electrical and electronic, exterior, general small parts, and interior. Each figure contrasts 2018 (panel A) and 2024 (panel B) for a single component category. The horizontal axis is on a logarithmic scale. As in the main text, blue dots mark the top 10 OEMs by transaction volume and red dots highlight Tesla and Tesla China as a benchmark of an independent procurement strategy.

Several patterns are consistent across categories. First, Japanese OEMs (Toyota, Nissan, Honda) consistently occupy the upper-right quadrant (high volume, high sharing ratio), reflecting their reliance on a broadly shared Tier-1 supplier base. Second, Tesla maintains a relatively low sharing ratio across all categories in both years. Third, categories tied to mature mechanical architectures (body, chassis, driveline) display tighter clustering of OEMs in the upper half of the sharing-ratio axis than electronics-intensive categories, indicating a more standardized supplier ecosystem for legacy subsystems. These observations reinforce the narrative in the main text that the fragmentation visible at the aggregate network level is driven chiefly by the electrification-sensitive categories rather than by the mechanical subsystems that remain comparatively stable.

4 CATEGORY-SPECIFIC NETWORK STRUCTURES

Figures S8–S14 present the OEM interdependency networks for each of the seven component categories not shown in the main text, comparing 2018 (left panel) with 2024 (right panel). Communities are detected by the greedy modularity-maximization (CNM) algorithm, and nodes are colored following the same convention as the main-text network figures: a node's hue indicates the dominant headquarters region of its community (red: Japan; blue: China; green: Europe; purple: Korea; orange: North America), and where a region contains more than one community, shade distinguishes them (darker = larger). The top 25 OEMs by weighted degree are labeled. Isolated nodes—OEMs that did not record any supplier sharing with other OEMs in a given category and year—are omitted for visual clarity.

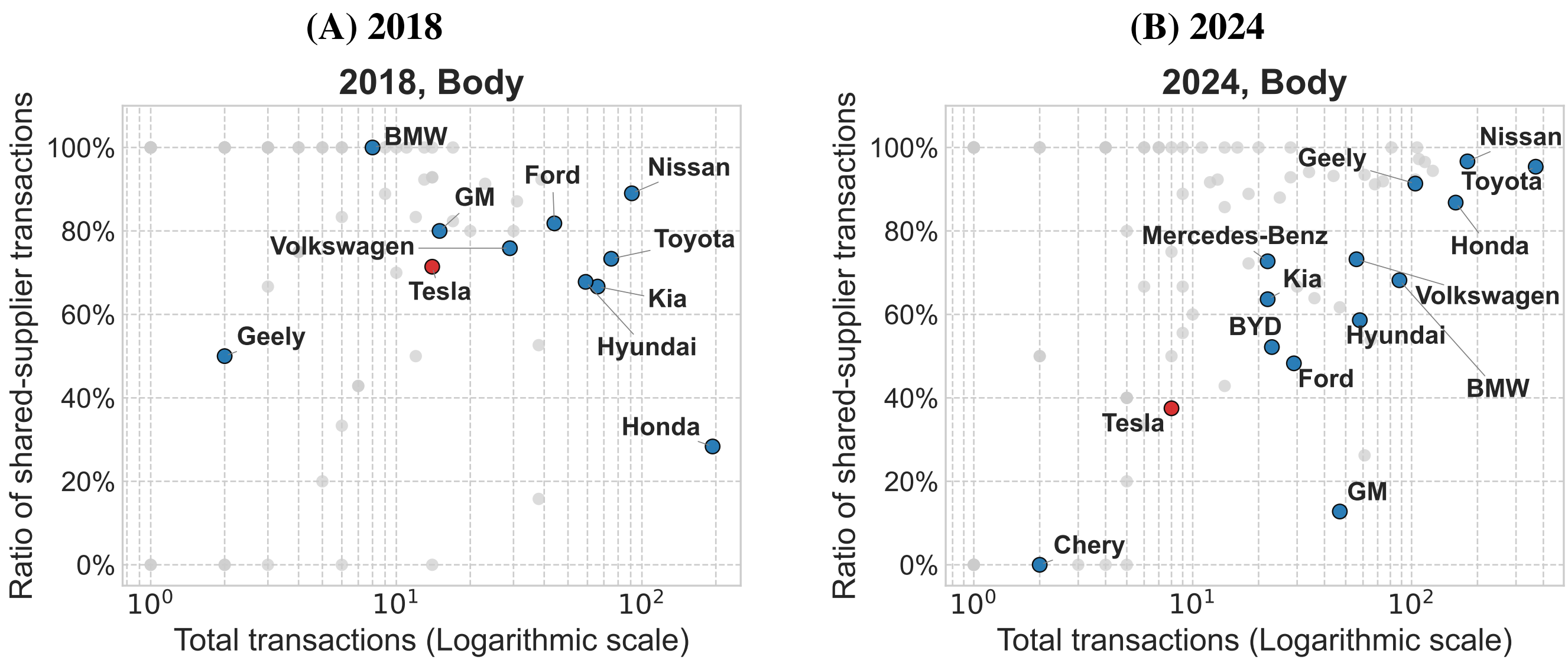

Figure S1. Procurement scatter plot for the body category in **(A)** 2018 and **(B)** 2024.

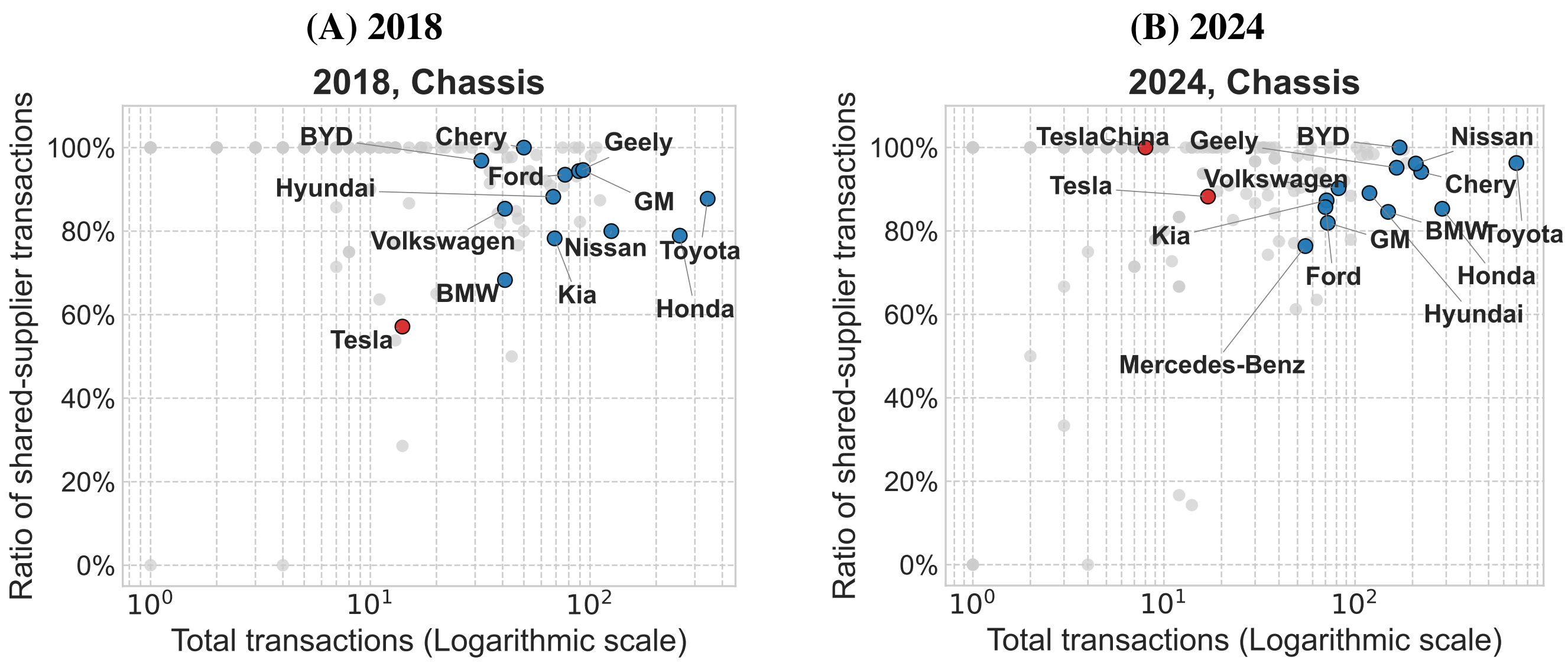

Figure S2. Procurement scatter plot for the chassis category in **(A)** 2018 and **(B)** 2024.

The category-level networks reveal several nuanced patterns that complement the aggregate view. Body, chassis, and exterior networks (Figures S8, S9, S12) remain relatively stable between 2018 and 2024, maintaining a core set of Japanese and European communities consistent with the maturity of these mechanical subsystems. Driveline and general small parts (Figures S10, S13) show moderate restructuring. In contrast, the electrical and electronic network (Figure S11) exhibits the most pronounced densification and re-ordering of any non-powertrain category, driven by the proliferation of electronic control units, sensors, and battery management systems associated with vehicle electrification and the software-defined vehicle. The interior network (Figure S14) shows a smaller but still notable shift toward tighter integration between Chinese and incumbent OEMs by 2024.

Taken together, these category-level networks confirm that the divergent paths documented in the main text for ICE-powertrain versus e-powertrain are not simply binary. They sit along a gradient whose shape is

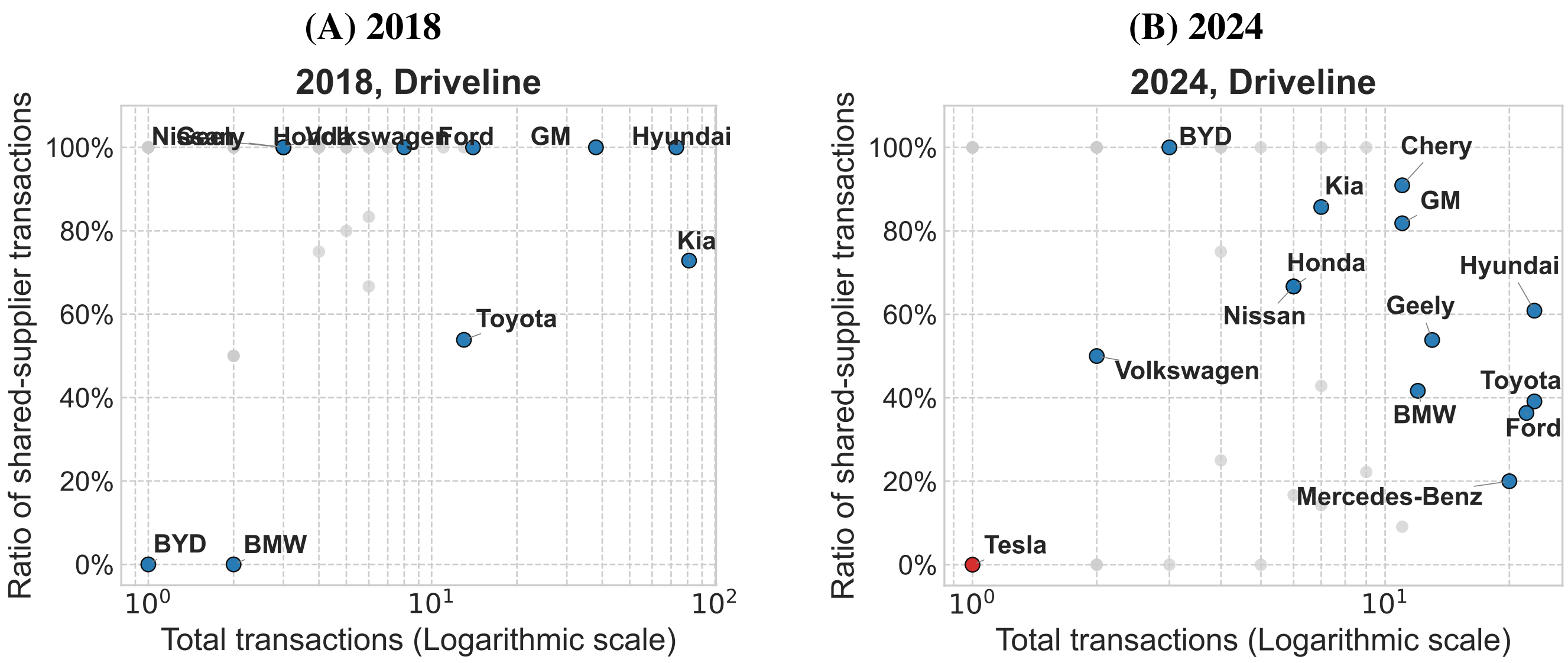

Figure S3. Procurement scatter plot for the driveline category in **(A)** 2018 and **(B)** 2024.

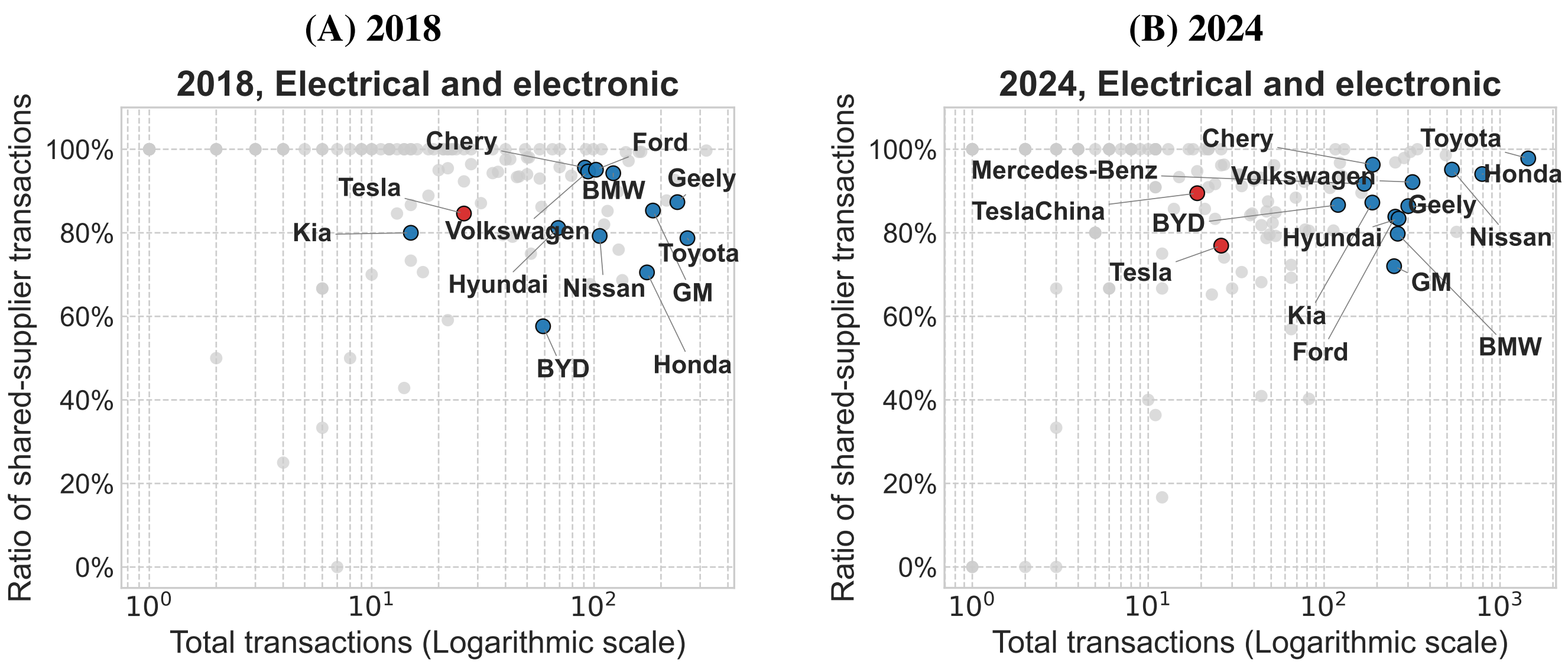

Figure S4. Procurement scatter plot for the electrical and electronic category in **(A)** 2018 and **(B)** 2024.

shaped by (i) the mechanical vs. electronic nature of each category and (ii) the degree of technological disruption affecting each subsystem under electrification.

5 COMMUNITY DETECTION RESULTS FOR ALL CATEGORIES

Tables S2–S19 report the community detection results for all nine component categories in both 2018 and 2024. Each table lists communities with two or more members. The “Top 5 Makers” column ranks member OEMs by their weighted degree in the Maker–Maker interdependency network (the total number of shared suppliers); the number in parentheses is that weighted degree. Group HQ Country Ratio and Maker HQ Country Ratio indicate the share of community members belonging to each country; only ratios of 20% or higher are shown for readability. Community indices within each table are ordered by community size (largest first).

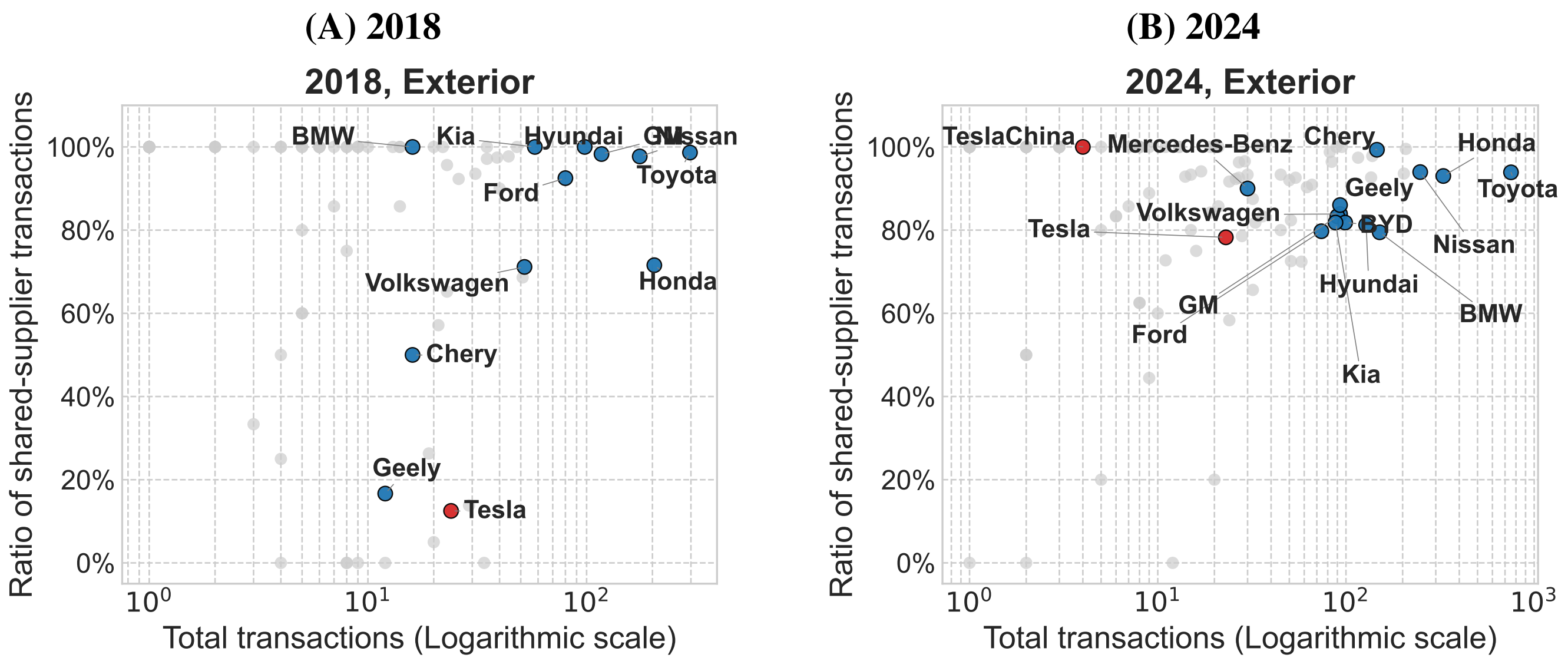

Figure S5. Procurement scatter plot for the exterior category in **(A)** 2018 and **(B)** 2024.

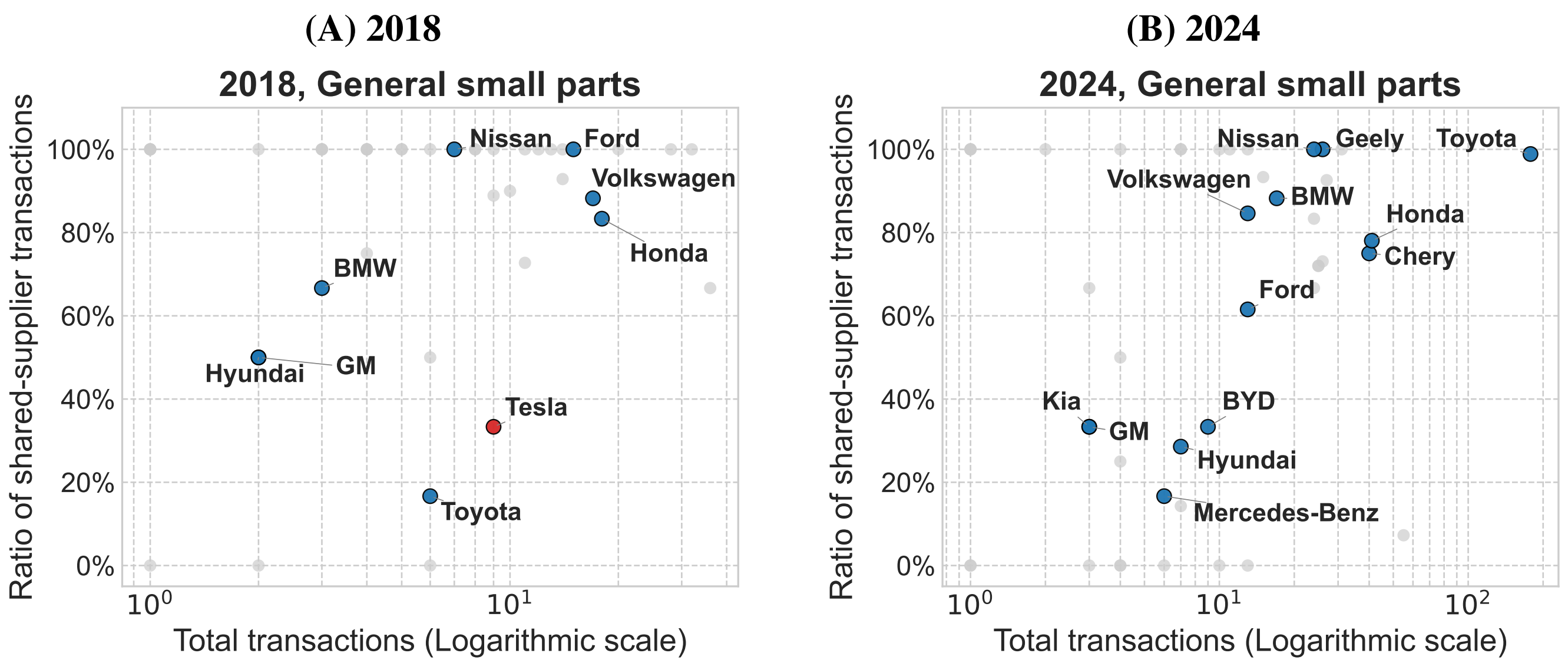

Figure S6. Procurement scatter plot for the general small parts category in **(A)** 2018 and **(B)** 2024.

Three cross-cutting observations emerge from the comprehensive tabulation. First, for categories associated with mature mechanical architectures (body, chassis, driveline, general small parts), community composition remains relatively stable between 2018 and 2024, with Japanese, European, and Chinese blocs persisting in recognizable form. Second, the ICE-powertrain tables (Tables S8 and S17) show a consolidation into more geographically cohesive communities by 2024—consistent with the main-text finding that legacy subsystems reinforce regional boundaries as investment migrates toward electrification. Third, the e-powertrain tables (Tables S10 and S19) illustrate the rapid densification of an entirely new ecosystem, with the 2024 partition featuring a Chinese-centric community, a European–Korean community, and a Japanese-led community that includes BYD—structures that were largely absent in 2018.

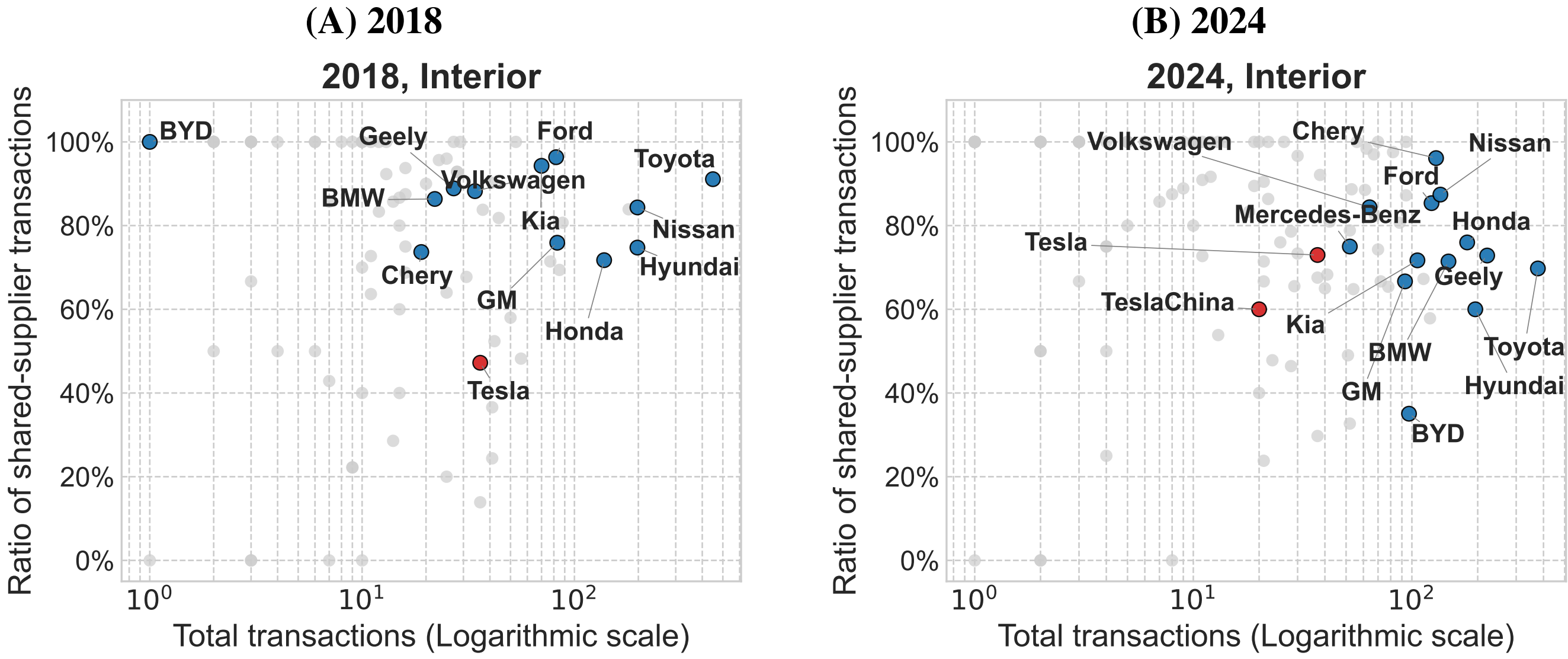

Figure S7. Procurement scatter plot for the interior category in **(A)** 2018 and **(B)** 2024.

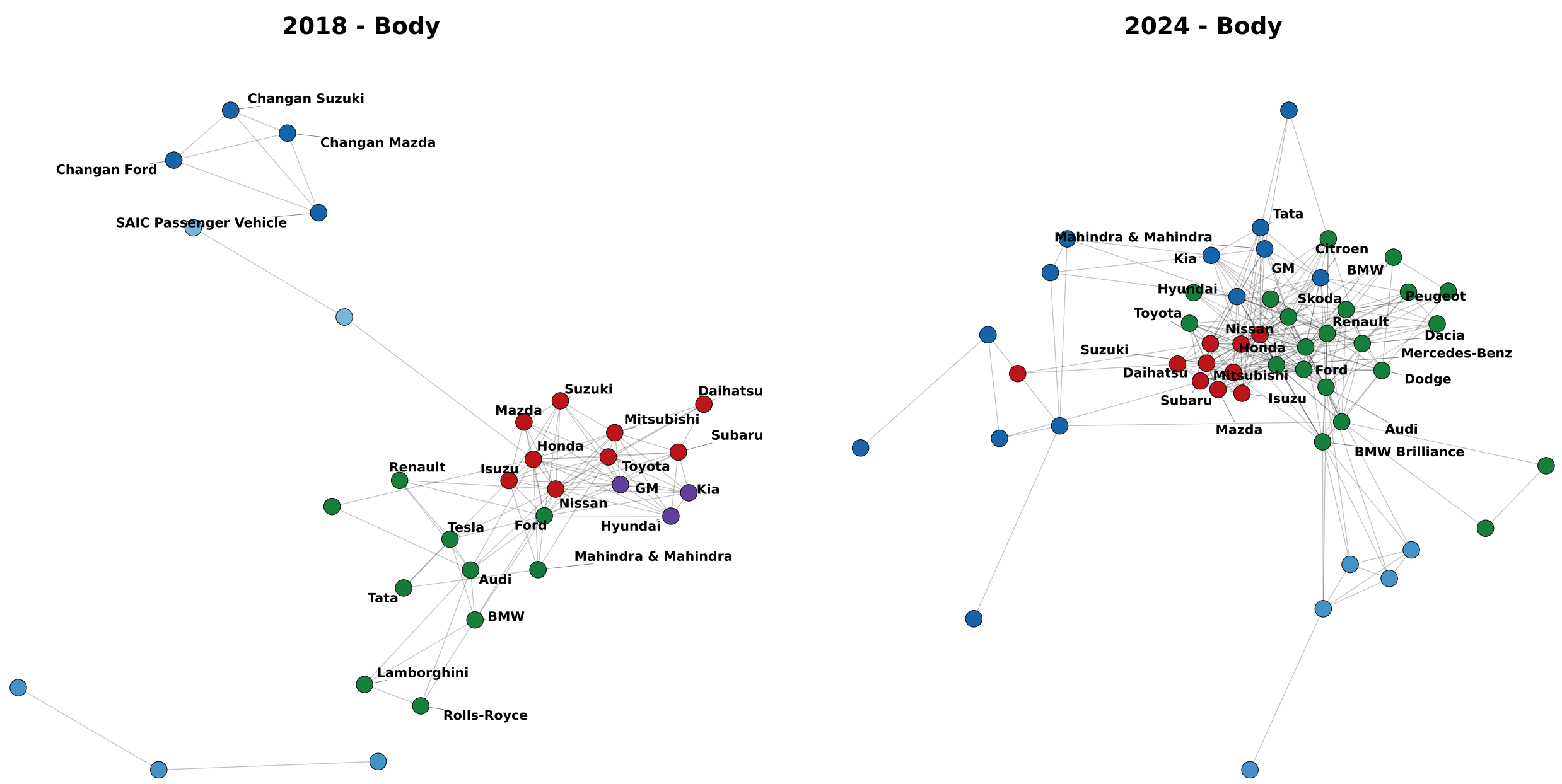

Figure S8. OEM interdependency network for body components, 2018 vs. 2024.

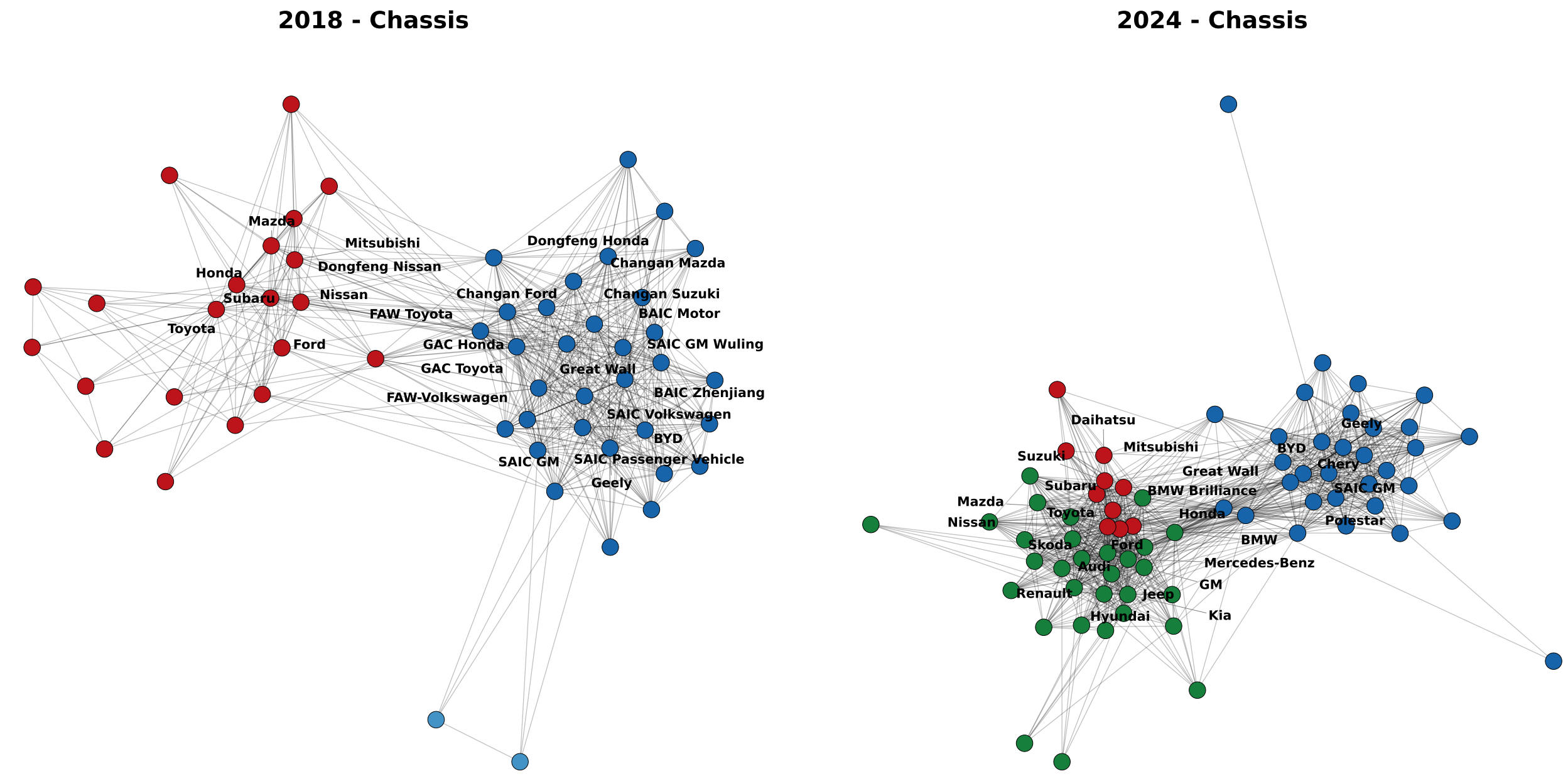

Figure S9. OEM interdependency network for chassis components, 2018 vs. 2024.

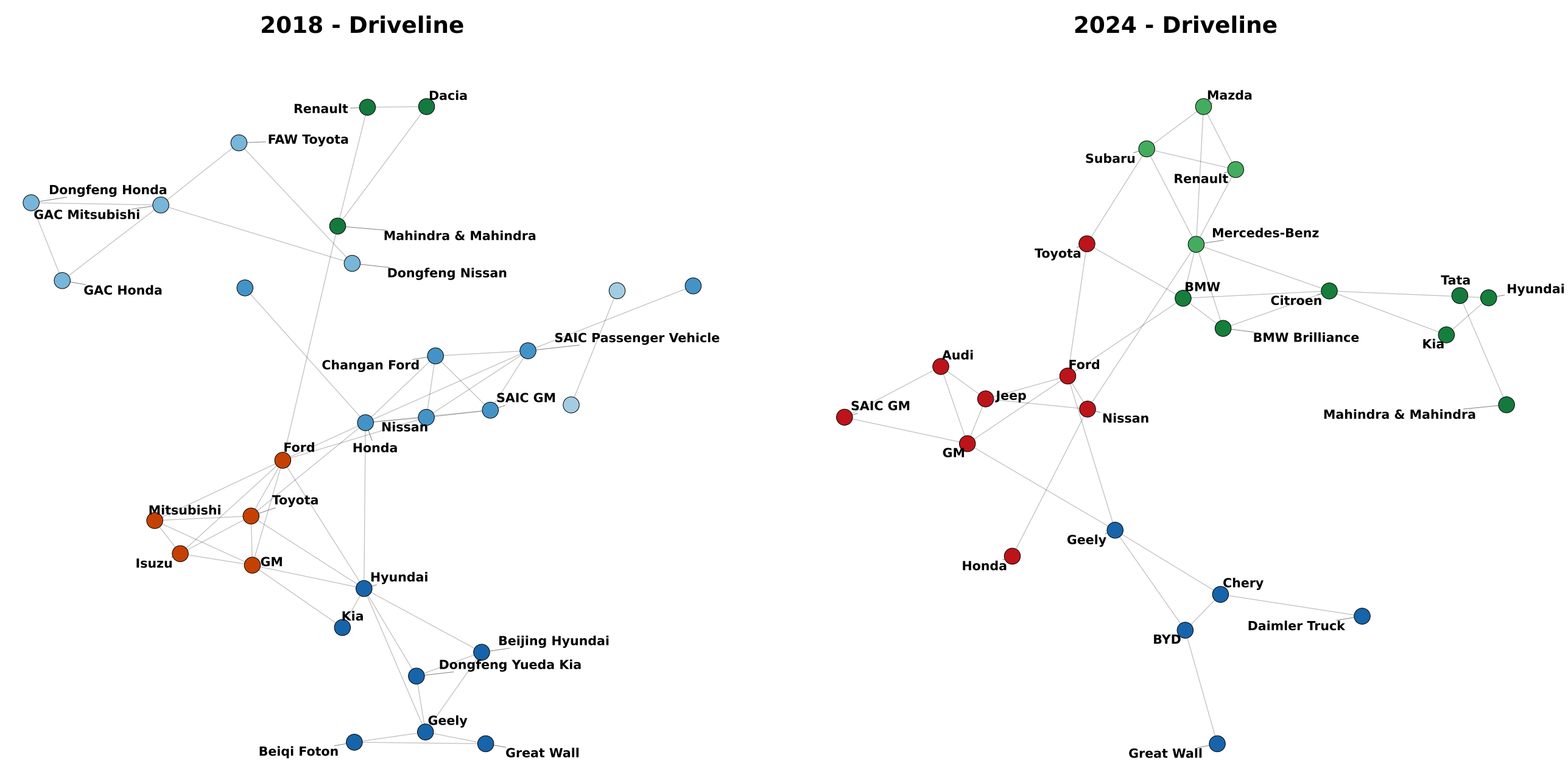

Figure S10. OEM interdependency network for driveline components, 2018 vs. 2024.

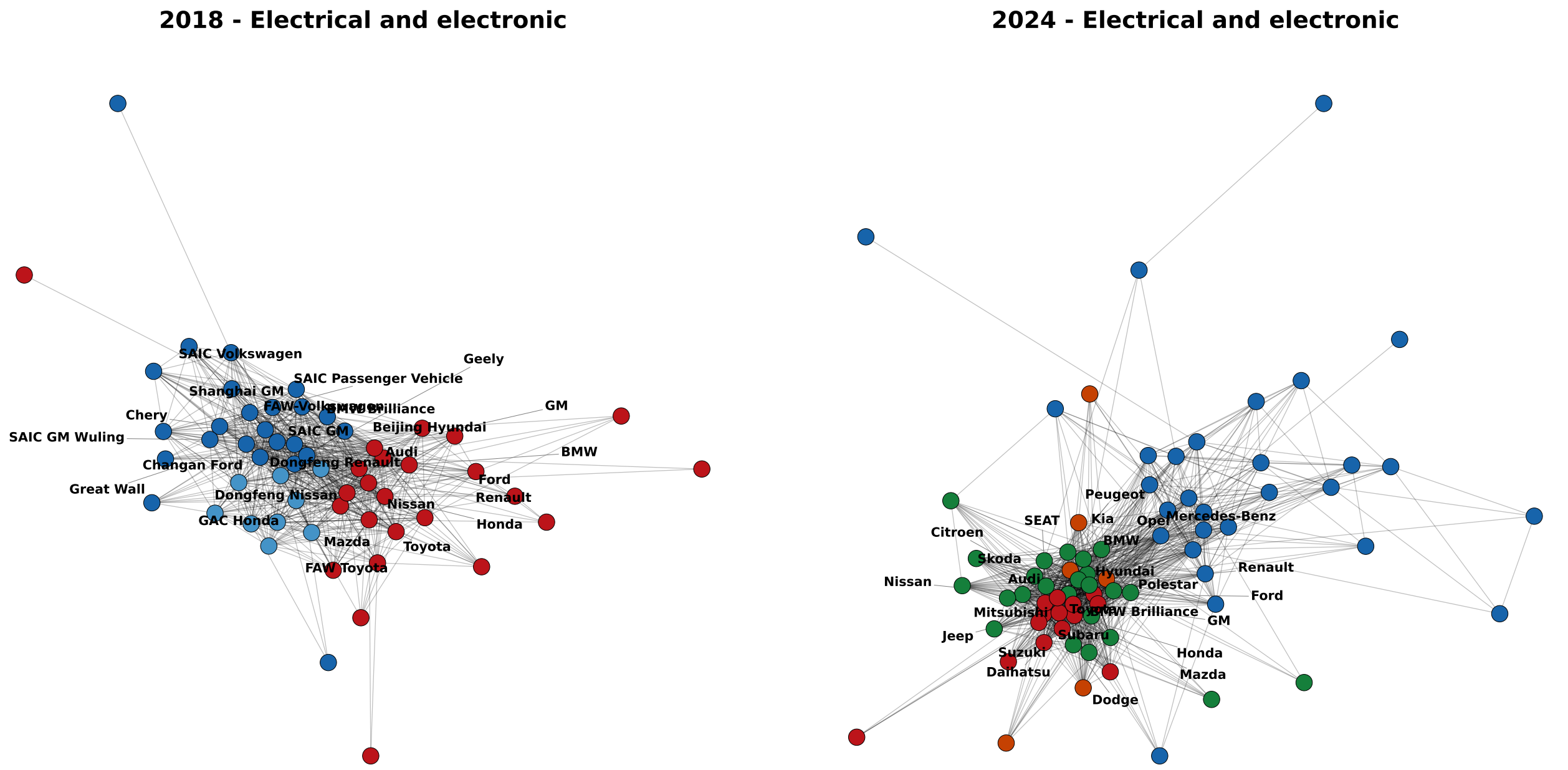

Figure S11. OEM interdependency network for electrical and electronic components, 2018 vs. 2024.

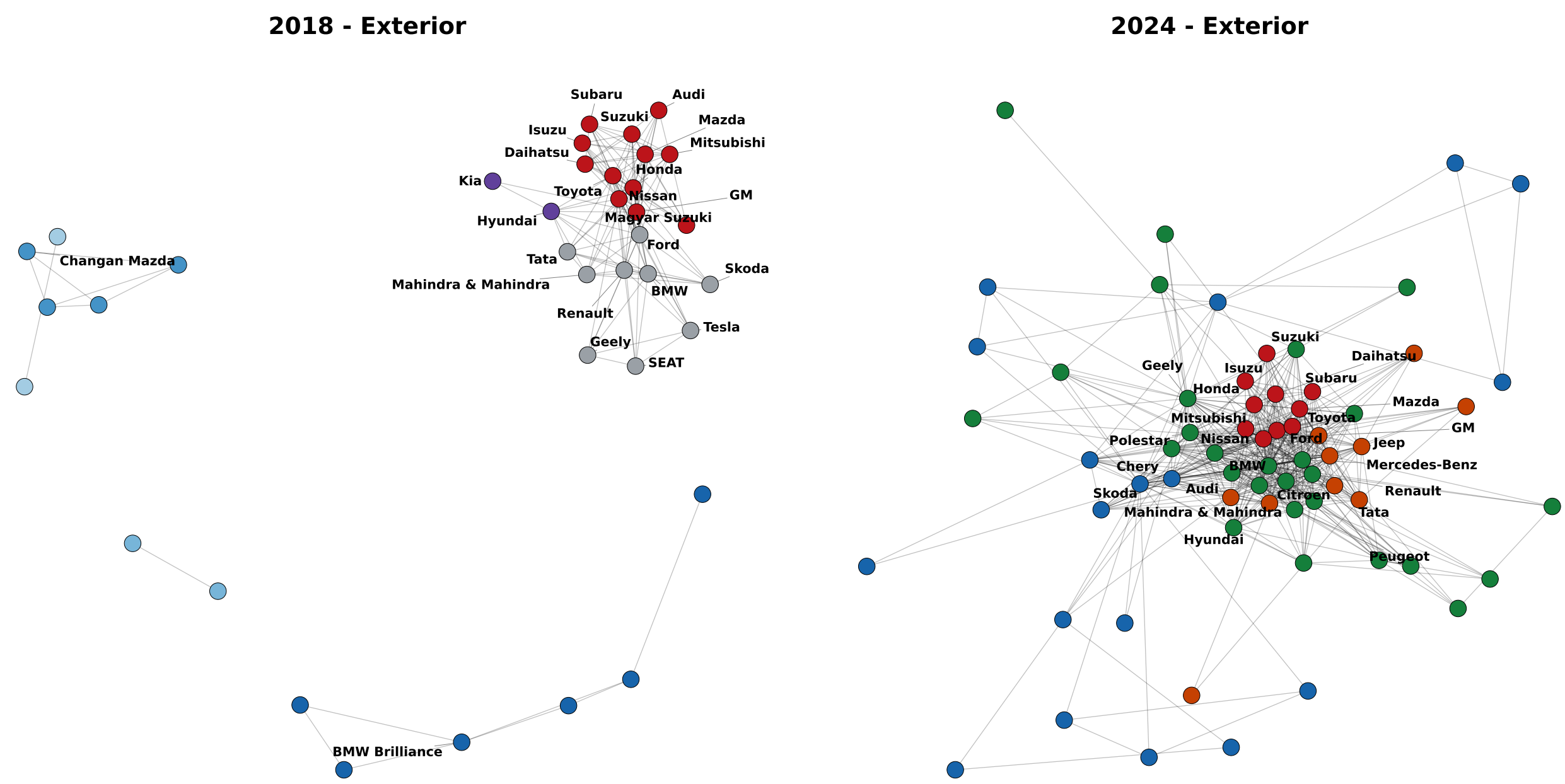

Figure S12. OEM interdependency network for exterior components, 2018 vs. 2024.

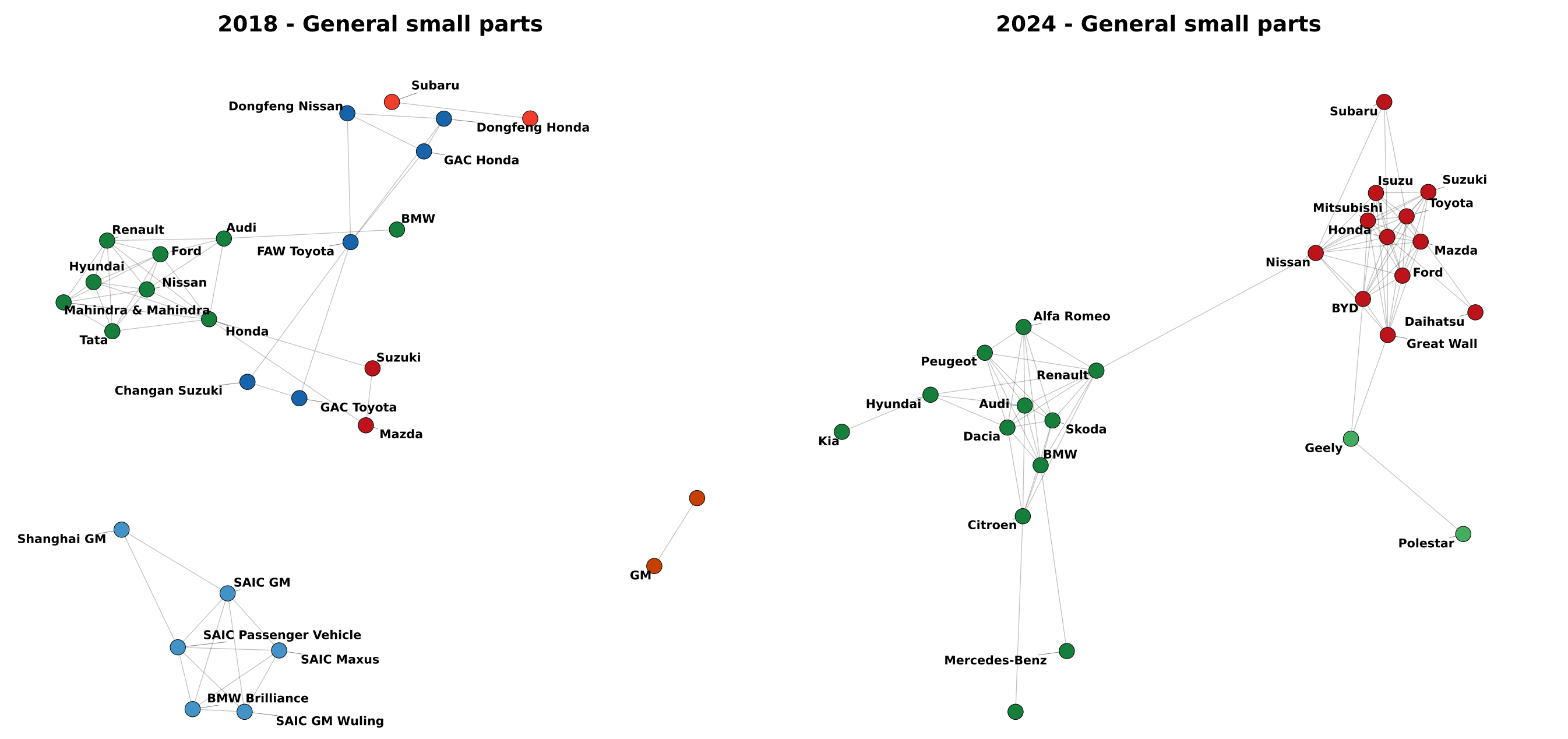

Figure S13. OEM interdependency network for general small parts, 2018 vs. 2024.

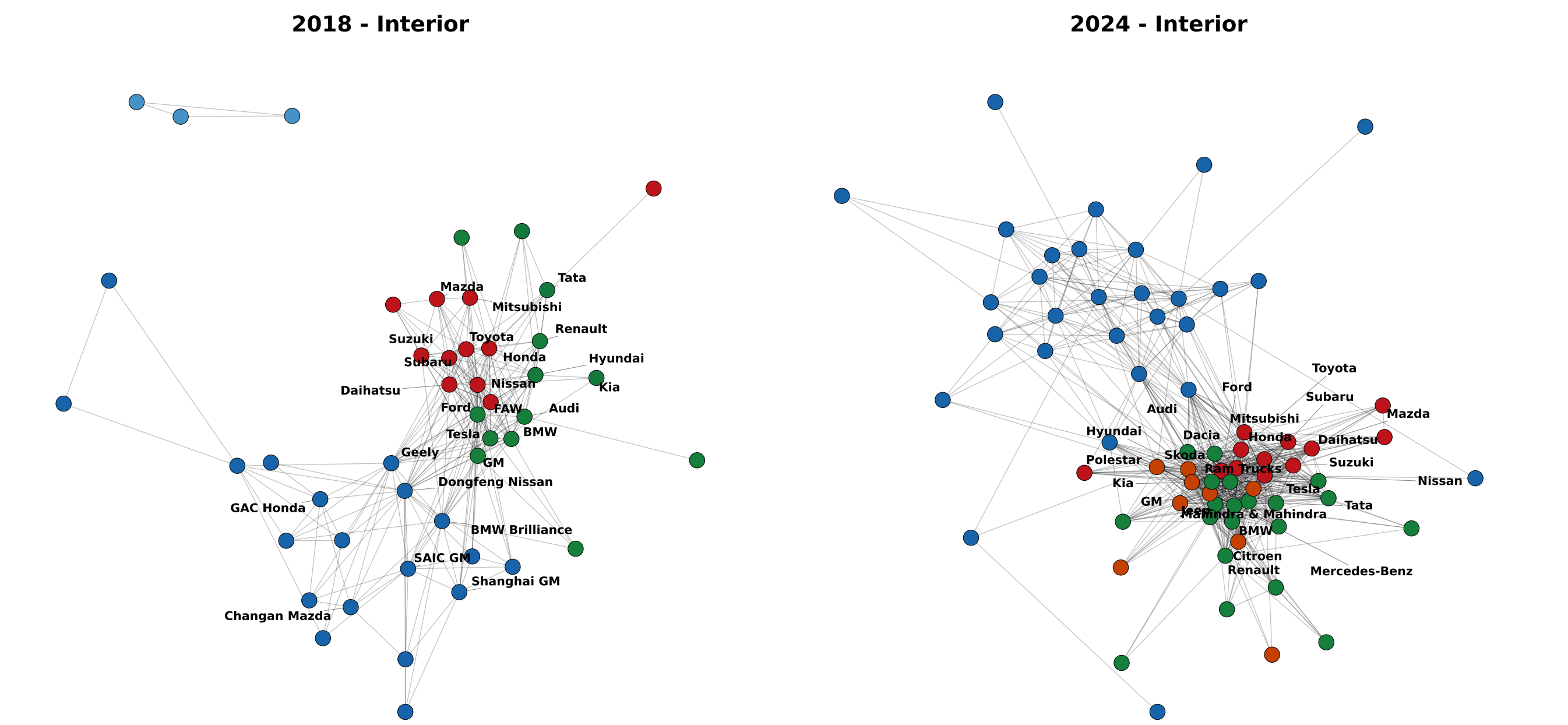

Figure S14. OEM interdependency network for interior components, 2018 vs. 2024.

Table S2. Community features for Body in 2018. Communities with size ≥ 2 are listed. Only HQ country ratios $\geq 20\%$ are shown.

Index (Size)	Top 5 Makers (by Total Shared Suppliers)	Group HQ Country Ratio	Maker HQ Country Ratio
1 (9)	Nissan (32), Toyota (25), Honda (23), Mitsubishi (15), Isuzu (15)	Japan (1.00)	Thailand (0.33), Japan (0.33)
2 (8)	Ford (29), Audi (22), BMW (11), Tesla (10), Renault (5)	Germany (0.50), USA (0.25)	USA (0.25), Germany (0.25)
3 (4)	Changan Mazda (3), SAIC Passenger Vehicle (3), Changan Suzuki (3), Changan Ford (3)	Japan (0.50), China (0.25), USA (0.25)	China (1.00)
4 (3)	Dongfeng Honda (2), GAC Mitsubishi (1), GAC Honda (1)	Japan (1.00)	China (1.00)
5 (3)	GM (17), Hyundai (16), Kia (16)	South Korea (0.67), USA (0.33)	Korea (0.67), Thailand (0.33)
6 (2)	GAC Toyota (2), Dongfeng Nissan (1)	Japan (1.00)	China (1.00)
7 (2)	Mahindra & Mahindra (6), Tata (4)	India (1.00)	India (1.00)

Table S3. Community features for Chassis in 2018. Communities with size ≥ 2 are listed. Only HQ country ratios $\geq 20\%$ are shown.

Index (Size)	Top 5 Makers (by Total Shared Suppliers)	Group HQ Country Ratio	Maker HQ Country Ratio
1 (32)	GAC Honda (77), SAIC GM (72), FAW Toyota (69), Dongfeng Nissan (69), FAW-Volkswagen (62)	China (0.37), Japan (0.33)	China (1.00)
2 (21)	Nissan (90), Honda (77), Toyota (74), Ford (66), Mitsubishi (62)	Japan (0.39)	Japan (0.29)
3 (2)	Dongfeng Yueda Kia (5), Beijing Hyundai (5)	South Korea (1.00)	China (1.00)

Table S4. Community features for Driveline in 2018. Communities with size ≥ 2 are listed. Only HQ country ratios $\geq 20\%$ are shown.

Index (Size)	Top 5 Makers (by Total Shared Suppliers)	Group HQ Country Ratio	Maker HQ Country Ratio
1 (7)	Hyundai (11), Geely (5), Kia (4), Dongfeng Yueda Kia (4), Beijing Hyundai (3)	South Korea (0.67), China (0.33)	China (0.71), Korea (0.29)
2 (7)	Honda (8), SAIC Passenger Vehicle (5), Nissan (5), SAIC GM (4), Changan Ford (4)	Germany (0.29), USA (0.29), Japan (0.29)	China (0.57)
3 (5)	GAC Mitsubishi (4), FAW Toyota (2), GAC Honda (2), Dongfeng Nissan (2), Dongfeng Honda (2)	Japan (1.00)	China (1.00)
4 (5)	Ford (10), Toyota (8), GM (7), Isuzu (6), Mitsubishi (5)	USA (0.67), Japan (0.33)	Thailand (0.60), India (0.20), USA (0.20)
5 (3)	Mahindra & Mahindra (3), Dacia (2), Renault (2)	France (0.67), India (0.33)	India (0.67), France (0.33)
6 (2)	Shanghai GM (1), FAW-Volkswagen (1)	USA (0.50), Germany (0.50)	China (1.00)

Table S5. Community features for Electrical and electronic in 2018. Communities with size ≥ 2 are listed. Only HQ country ratios $\geq 20\%$ are shown.

Index (Size)	Top 5 Makers (by Total Shared Suppliers)	Group HQ Country Ratio	Maker HQ Country Ratio
1 (24)	Geely (165), SAIC GM (138), Great Wall (117), Changan Ford (111), FAW-Volkswagen (100)	China (0.45), USA (0.23)	China (1.00)
2 (24)	Nissan (146), Honda (127), Ford (121), Audi (107), BMW (87)	Japan (0.30), USA (0.20), Germany (0.20)	Japan (0.21)
3 (9)	Dongfeng Nissan (146), GAC Honda (74), Dongfeng Renault (64), FAW Toyota (62), Zhengzhou Nissan (54)	Japan (0.89)	China (1.00)

Table S6. Community features for Exterior in 2018. Communities with size ≥ 2 are listed. Only HQ country ratios $\geq 20\%$ are shown.

Index (Size)	Top 5 Makers (by Total Shared Suppliers)	Group HQ Country Ratio	Maker HQ Country Ratio
1 (12)	Honda (82), Nissan (75), Toyota (59), Mazda (44), Suzuki (36)	Japan (0.78)	Thailand (0.42), Japan (0.25)
2 (9)	Ford (34), BMW (21), Renault (19), Mahindra & Mahindra (13), Tata (13)	Germany (0.33), USA (0.22), India (0.22)	India (0.44)
3 (6)	BMW Brilliance (4), Dongfeng Nissan (3), Chery (2), SAIC Volkswagen (2), FAW-Volkswagen (2)	Germany (0.50)	China (1.00)
4 (4)	Changan Mazda (3), FAW Car (3), FAW (3), FAW Mazda (3)	Japan (1.00)	China (1.00)
5 (2)	Dongfeng Yueda Kia (1), Beijing Hyundai (1)	South Korea (1.00)	China (1.00)
6 (2)	Dongfeng Honda (1), GAC Honda (1)	Japan (1.00)	China (1.00)
7 (2)	Hyundai (22), Kia (9)	South Korea (1.00)	Korea (1.00)

Table S7. Community features for General small parts in 2018. Communities with size ≥ 2 are listed. Only HQ country ratios $\geq 20\%$ are shown.

Index (Size)	Top 5 Makers (by Total Shared Suppliers)	Group HQ Country Ratio	Maker HQ Country Ratio
1 (9)	Ford (14), Honda (11), Nissan (9), Audi (9), Renault (8)	India (0.22), Japan (0.22), Germany (0.22)	India (0.67)
2 (6)	GAC Honda (5), Dongfeng Honda (5), FAW Toyota (5), Dongfeng Nissan (3), Changan Suzuki (2)	Japan (1.00)	China (1.00)
3 (6)	SAIC Passenger Vehicle (6), SAIC GM (6), SAIC GM Wuling (4), SAIC Maxus (4), BMW Brilliance (4)	USA (0.50), China (0.33)	China (1.00)
4 (2)	Mazda (2), Suzuki (2)	Japan (1.00)	Thailand (1.00)
5 (2)	Tesla (1), GM (1)	USA (1.00)	USA (0.50), Canada (0.50)
6 (2)	Toyota (1), Subaru (1)	Japan (1.00)	USA (1.00)

Table S8. Community features for ICE-powertrain in 2018. Communities with size $\geq$ 2 are listed. Only HQ country ratios $\geq$ 20% are shown.

Index (Size)	Top 5 Makers (by Total Shared Suppliers)	Group HQ Country Ratio	Maker HQ Country Ratio
1 (21)	Ford (162), Nissan (152), BMW Brilliance (152), BMW (115), Audi (114)	Japan (0.29), Germany (0.29)	–
2 (20)	SAIC GM (219), FAW-Volkswagen (152), Changan Ford (150), SAIC Passenger Vehicle (150), SAIC Volkswagen (150)	China (0.53), USA (0.26)	China (1.00)
3 (16)	Dongfeng Nissan (274), GAC Honda (174), GAC Toyota (172), FAW Toyota (169), Toyota (163)	Japan (0.87)	China (0.81)

Table S9. Community features for Interior in 2018. Communities with size $\geq$ 2 are listed. Only HQ country ratios $\geq$ 20% are shown.

Index (Size)	Top 5 Makers (by Total Shared Suppliers)	Group HQ Country Ratio	Maker HQ Country Ratio
1 (19)	Dongfeng Nissan (39), Geely (26), BMW Brilliance (21), GAC Honda (16), SAIC GM (16)	China (0.28), Japan (0.22), USA (0.22)	China (1.00)
2 (11)	Nissan (76), Toyota (59), Honda (52), Daihatsu (43), Subaru (40)	Japan (1.00)	Thailand (0.45), Japan (0.36)
3 (9)	Ford (62), GM (37), Audi (31), BMW (28), Tesla (27)	USA (0.33), Germany (0.33), France (0.22)	USA (0.22)
4 (4)	Hyundai (32), Tata (12), Kia (12), Mahindra & Mahindra (7)	India (0.50), South Korea (0.50)	India (0.50), Korea (0.50)
5 (3)	FAW Toyota (2), Changan Suzuki (2), GAC Toyota (2)	Japan (1.00)	China (1.00)

Table S10. Community features for e-powertrain in 2018. Communities with size $\geq$ 2 are listed. Only HQ country ratios $\geq$ 20% are shown.

Index (Size)	Top 5 Makers (by Total Shared Suppliers)	Group HQ Country Ratio	Maker HQ Country Ratio
1 (10)	Geely (8), BAIC Motor (7), Chery (6), Great Wall (6), Changhe Suzuki (5)	China (0.78)	China (1.00)
2 (7)	Dongfeng Nissan (11), Nissan (8), GM (6), Suzuki (5), SAIC GM (5)	Japan (0.57), USA (0.43)	Japan (0.43), USA (0.29), China (0.29)
3 (2)	BMW (1), BMW Brilliance (1)	Germany (1.00)	Germany (0.50), China (0.50)
4 (2)	Ford (3), Changan Ford (2)	USA (1.00)	USA (0.50), China (0.50)
5 (2)	FAW Toyota (1), GAC Toyota (1)	Japan (1.00)	China (1.00)
6 (2)	Dongfeng Yueda Kia (1), Hyundai (1)	South Korea (1.00)	China (0.50), Korea (0.50)

Table S11. Community features for Body in 2024. Communities with size $\geq$ 2 are listed. Only HQ country ratios $\geq$ 20% are shown.

Index (Size)	Top 5 Makers (by Total Shared Suppliers)	Group HQ Country Ratio	Maker HQ Country Ratio
1 (21)	BMW (74), Renault (49), Audi (43), Mercedes-Benz (39), Skoda (37)	Germany (0.33), Netherlands (0.24)	China (0.24), Germany (0.24)
2 (13)	Hyundai (35), Citroen (27), Mahindra & Mahindra (25), Tata (25), Kia (15)	Japan (0.25)	China (0.54), India (0.38)
3 (10)	Nissan (103), Toyota (81), Mitsubishi (72), Honda (64), Suzuki (59)	Japan (1.00)	Japan (0.70)
4 (5)	SAIC Volkswagen (6), FAW-Volkswagen (5), Beijing Benz (5), SAIC Passenger Vehicle (5), SAIC GM (1)	Germany (0.60), China (0.20), USA (0.20)	China (1.00)

Table S12. Community features for Chassis in 2024. Communities with size $\geq$ 2 are listed. Only HQ country ratios $\geq$ 20% are shown.

Index (Size)	Top 5 Makers (by Total Shared Suppliers)	Group HQ Country Ratio	Maker HQ Country Ratio
1 (32)	Chery (135), Geely (126), BYD (118), BMW Brilliance (104), Polestar (98)	China (0.39)	China (1.00)
2 (30)	BMW (267), Mercedes-Benz (131), GM (128), Ford (126), Audi (118)	Netherlands (0.40), Germany (0.23)	–
3 (10)	Toyota (341), Nissan (309), Honda (297), Mazda (219), Mitsubishi (175)	Japan (1.00)	Japan (0.60), Thailand (0.20)

Table S13. Community features for Driveline in 2024. Communities with size $\geq$ 2 are listed. Only HQ country ratios $\geq$ 20% are shown.

Index (Size)	Top 5 Makers (by Total Shared Suppliers)	Group HQ Country Ratio	Maker HQ Country Ratio
1 (8)	GM (7), Ford (6), Jeep (5), Nissan (4), Audi (4)	USA (0.38), Japan (0.38)	USA (0.50), Japan (0.25)
2 (5)	Chery (5), Geely (5), BYD (4), Daimler Truck (1), Great Wall (1)	China (0.75), Germany (0.25)	China (0.80), Germany (0.20)
3 (5)	BMW (5), Citroen (5), Hyundai (4), Kia (4), BMW Brilliance (3)	South Korea (0.40), Germany (0.40), Netherlands (0.20)	Korea (0.40), Germany (0.20), Spain (0.20), China (0.20)
4 (4)	Mercedes-Benz (7), Subaru (4), Mazda (3), Renault (3)	Japan (0.33), France (0.33), Germany (0.33)	Japan (0.50), France (0.25), India (0.25)
5 (2)	Mahindra & Mahindra (1), Tata (1)	India (1.00)	India (1.00)

Table S14. Community features for Electrical and electronic in 2024. Communities with size $\geq$ 2 are listed. Only HQ country ratios $\geq$ 20% are shown.

Index (Size)	Top 5 Makers (by Total Shared Suppliers)	Group HQ Country Ratio	Maker HQ Country Ratio
1 (29)	Geely (149), Great Wall (107), Chery (89), BYD (86), SAIC Passenger Vehicle (70)	China (0.41), Japan (0.22)	China (1.00)
2 (24)	Renault (373), BMW (358), Audi (355), Mercedes-Benz (344), Skoda (274)	Netherlands (0.33), Germany (0.33)	–
3 (14)	Honda (518), Nissan (504), Toyota (434), Mitsubishi (421), Mazda (414)	Japan (0.64)	USA (0.29), Japan (0.29), Thailand (0.21)
4 (6)	Hyundai (287), Kia (270), Tesla (56), Chrysler (46), Rolls-Royce (10)	Germany (0.33), South Korea (0.33)	India (0.33)

Table S15. Community features for Exterior in 2024. Communities with size $\geq$ 2 are listed. Only HQ country ratios $\geq$ 20% are shown.

Index (Size)	Top 5 Makers (by Total Shared Suppliers)	Group HQ Country Ratio	Maker HQ Country Ratio
1 (27)	BMW (140), Audi (101), Renault (95), Citroen (71), Skoda (69)	Germany (0.37), Netherlands (0.33)	China (0.37)
2 (18)	Chery (48), Great Wall (42), BYD (23), Changan Ford (14), BAIC Motor (12)	China (0.41), Japan (0.24)	China (1.00)
3 (10)	GM (91), Ford (90), Hyundai (69), Jeep (66), Tata (53)	Netherlands (0.30), USA (0.20), South Korea (0.20), India (0.20)	India (0.50), Mexico (0.20)
4 (10)	Nissan (243), Toyota (214), Honda (197), Mitsubishi (141), Mazda (136)	Japan (1.00)	Thailand (0.60), Japan (0.40)

Table S16. Community features for General small parts in 2024. Communities with size $\geq$ 2 are listed. Only HQ country ratios $\geq$ 20% are shown.

Index (Size)	Top 5 Makers (by Total Shared Suppliers)	Group HQ Country Ratio	Maker HQ Country Ratio
1 (12)	Audi (22), Dacia (19), Skoda (18), Renault (17), BMW (17)	Germany (0.33), Netherlands (0.25)	Germany (0.25)
2 (12)	Toyota (35), Honda (27), Mitsubishi (27), Isuzu (25), Mazda (23)	Japan (0.75)	Thailand (0.58)
3 (2)	Geely (4), Polestar (1)	China (1.00)	China (1.00)

Table S17. Community features for ICE-powertrain in 2024. Communities with size $\geq$ 2 are listed. Only HQ country ratios $\geq$ 20% are shown.

Index (Size)	Top 5 Makers (by Total Shared Suppliers)	Group HQ Country Ratio	Maker HQ Country Ratio
1 (16)	Audi (90), BMW (81), Skoda (81), Mercedes-Benz (75), GM (70)	Netherlands (0.44), Germany (0.31)	USA (0.25)
2 (13)	SAIC Passenger Vehicle (16), BYD (16), Chery (15), Great Wall (13), Geely (12)	China (0.42), USA (0.33)	China (1.00)
3 (11)	Kia (51), Renault (45), Mahindra & Mahindra (39), Hyundai (36), Citroen (34)	Netherlands (0.36), South Korea (0.27)	India (0.36)
4 (10)	Toyota (208), Nissan (199), Mitsubishi (163), Honda (155), Mazda (122)	Japan (1.00)	Japan (0.80), Thailand (0.20)

Table S18. Community features for Interior in 2024. Communities with size $\geq$ 2 are listed. Only HQ country ratios $\geq$ 20% are shown.

Index (Size)	Top 5 Makers (by Total Shared Suppliers)	Group HQ Country Ratio	Maker HQ Country Ratio
1 (29)	BYD (39), Tesla China (34), Geely (31), Chery (30), BMW Brilliance (30)	China (0.32), Germany (0.21), Japan (0.21)	China (0.93)
2 (20)	BMW (128), Skoda (125), Mercedes-Benz (107), Renault (92), Mahindra & Mahindra (86)	Netherlands (0.40), Germany (0.35)	Germany (0.25)
3 (12)	Toyota (189), Honda (180), Ford (156), Nissan (137), Mitsubishi (105)	Japan (0.78)	Japan (0.42), Thailand (0.33)
4 (9)	Hyundai (133), GM (126), Kia (109), Tesla (80), Jeep (69)	Netherlands (0.44), USA (0.22), South Korea (0.22)	India (0.33), Italy (0.22), USA (0.22)

Table S19. Community features for e-powertrain in 2024. Communities with size $\geq$ 2 are listed. Only HQ country ratios $\geq$ 20% are shown.

Index (Size)	Top 5 Makers (by Total Shared Suppliers)	Group HQ Country Ratio	Maker HQ Country Ratio
1 (22)	Geely (88), Chery (66), Great Wall (58), Tesla China (51), Polestar (49)	China (0.38)	China (0.91)
2 (20)	BMW (90), Mercedes-Benz (69), Peugeot (61), Ford (55), Citroen (51)	Netherlands (0.30), Germany (0.30)	Germany (0.20)
3 (14)	Toyota (41), Nissan (36), Mazda (29), BYD (29), Honda (22)	Japan (0.82)	Japan (0.57), China (0.21)
4 (9)	IM Motors (49), SAIC Maxus (45), SAIC GM (37), SAIC Volkswagen (23), SAIC Passenger Vehicle (18)	China (0.33), Germany (0.22), USA (0.22)	China (1.00)
